# Citrullination Accompanies the Development of Carotid Atherosclerotic Plaques

**DOI:** 10.2174/0109298673327175240929222308

**Published:** 2024-10-17

**Authors:** Anastasia Kanonykina, Elena Velikanova, Victoria Markova, Leo Bogdanov, Daria Shishkova, Amin Shabaev, Maxim Sinitsky, Anna Sinitskaya, Alyona Poddubnyak, Anastasia Lazebnaya, Alexander Stepanov, Arina Tyurina, Arseniy Lobov, Bozhana Zainullina, Arseniy Yuzhalin, Anton Kutikhin

**Affiliations:** 1 Department of Experimental Medicine, Research Institute for Complex Issues of Cardiovascular Diseases, 6 Sosnovy Boulevard, 650002, Kemerovo, Russia;; 2 Laboratory of Regenerative Biomedicine, Institute of Cytology of the RAS, 4 Tikhoretskiy Prospekt, St. Petersburg, 194064, Russia;; 3 Centre for Molecular and Cell Technologies, St. Petersburg State University, Universitetskaya Embankment, 7/9, 199034, St. Petersburg, Russia

**Keywords:** atherosclerosis, neointima, carotid artery, citrullination, neutrophils, PADI2, PADI4, peptidylcitrulline

## Abstract

**Background:**

Citrullination represents a post-translational modification primarily mediated by peptidylarginine deiminase (PADI) 2 and 4 and resulting in the conversion of positively charged peptidylarginine to neutrally charged peptidylcitrulline. Molecular consequences of citrullination include the generation of neoepitopes which provoke the production of autoantibodies implicated in the development of autoimmune diseases. As citrullination initiates, promotes, and is enhanced by aseptic inflammation which plays a pivotal role in atherosclerosis, we proposed that citrullination might accompany the development of atherosclerotic vascular disease.

**Objective:**

To investigate features and patterns of citrullination in atherosclerotic plaques.

**Methods:**

We collected carotid atherosclerotic plaques (n = 14) and adjacent arterial segments (n = 14) which were pairwise excised during the carotid endarterectomy. The tissues were examined employing proteomic profiling (ultra-high performance liquid chromatography-tandem mass spectrometry analysis), haematoxylin and eosin staining, Western blotting and immunofluorescence staining for peptidylcitrulline, PADI2, and PADI4, and gene expression analysis. To better explore the mechanisms of citrullination in the neointima, we have also stained excised plaques for the extracellular vesicle markers (CD9 and CD81) and assessed co-localisation of PADI2 (a citrullination marker) with CD81 (an extracellular vesicle marker). In order to study the systemic response to citrullination in an atherosclerotic vascular disease setting, we measured the level of anti-citrullinated protein antibodies in the serum of patients with ischaemic stroke and healthy volunteers.

**Results:**

Proteomic profiling found 213 plaque-specific and 111 intact arteria-specific proteins, as well as 46 proteins and 13 proteins which have been respectively upregulated or downregulated in plaques as compared with the adjacent intact segments. Among the top 20 upregulated proteins were atherogenic apolipoprotein B-100, iron-associated protein haptoglobin, and matrix metal-loproteinase-9, together indicating the advanced stage of plaque progression. In comparison with the intact arterial segments, plaques demonstrated protein signatures of innate immune response and oxidative stress, suggesting aseptic inflammation as a driver of atherosclerotic vascular disease. Both peptidylcitrulline and PADI2 have been abundant in the neointima but negligible in tunica media; further, the levels of peptidylcitrulline, PADI2, and PADI4 were elevated in plaque lysates in comparison with those from adjacent arterial segments (*p* = 0.025, 0.025, and 0.010, respectively). Notably, PADI2 and peptidylcitrulline were co-localised with the cells in the neointima and a considerable proportion of PADI2 was co-localised with CD81-positive extracellular vesicles (*p* = 0.003). Albeit citrullinated histone H3 and myeloperoxidase showed higher signal in the neointima than in tunica media (*p* = 0.048 and 0.023, respectively), we did not observe any signs of neutrophil extracellular traps (*e.g.,* unwound chromatin or co-localisation of citrullinated histone H3 with neutrophil elastase) in the plaque tissue. Serum anti-citrullinated protein antibodies were not elevated in patients with ischaemic stroke (*p* = 0.71), suggesting that vascular citrullination likely does not trigger a generalised immune response.

**Conclusion:**

The development of carotid atherosclerosis is associated with citrullination, although it represents a local rather than systemic phenomenon in this clinical scenario.

## 1 INTRODUCTION

Protein citrullination is a post-translational modification catalysed by enzymes known as peptidylarginine deiminases (PADIs), which are a family of 5 Ca^2+^-dependent enzymes (PADI1, PADI2, PADI3, PADI4, and PADI6) that catalyze the hydrolysis of guanidino group of the arginine side chain to an ureido group, resulting in the conversion of arginine to citrulline [1] (Fig. **S1**). Unlike positively charged peptidyl-arginine, peptidylcitrulline has a neutral charge; therefore, the biological significance of citrullination is altering protein hydrophobicity and conformation. In turn, conformational changes result in the emergence of neoepitopes inducing the generation of autoantibodies which provoke inflammation [2]. Citrullination has been implicated in rheumatoid arthritis, multiple sclerosis, psoriasis, and cancer [3-6]. The inflammatory response is a key trigger in the development of atherosclerosis as well as in the instability and rupture of the atherosclerotic plaque [7-9]. Pro-inflammatory endothelial activation impairs endothelial-dependent vasoactive mechanisms and provokes acute coronary events in patients with stable angina even without obstructive coronary stenosis and contributes to the plaque rupture, having been affected by epigenetic modifications [7-9].

Studies connecting citrullination and cardiovascular disease (CVD), including atherosclerosis, are sparse, and its role in the pathophysiology remains unclear [2]. One consequence of citrullination for cardiovascular pathophysiology is the generation of neutrophil extracellular traps (NETs), a process determined by citrullination of histones, ejection of uncondensed chromatin from neutrophils, and decoration of released chromatin with antimicrobial proteins such as neutrophil elastase (NE/ELA2) or myeloperoxidase (MPO). In the blood vessels, NETs act as a pro-thrombotic scaffold causing adhesion, activation, and aggregation of platelets and adsorbing blood coagulation factors [10-14], whereas administration of DNases [11, 13, 14] and heparin [14] or PAD4 knockout [12] can inhibit NETosis and thrombosis. NETs are involved in the development of deep vein thrombosis [10, 12-14], chronic thromboembolic pulmonary hypertension [11], ischemia-related coronary microvascular obstruction [15], atherosclerosis [16], and atherothrombosis [16]. Increased serum complexes of MPO and DNA (NET marker) were associated with atherosclerosis and an increased risk of major adverse cardiovascular events [17].

Citrullinated proteins and NETs have been detected in atherosclerotic plaques [18, 19], but the functional mechanisms remain elusive. NETosis has been implicated in the pathogenesis of atherosclerosis in hyperlipidemic mice [20], and the release of NETs by neutrophils primes macrophages for cytokine production in atherosclerotic plaques [21]. NETs were identified in the scaffolds of coronary thrombi and thrombotic NET burden correlated with infarct size [16]. Neutrophils and NETs correlated with ruptured or eroded but not intact plaques, having been found in thrombi and haemorrhages as well as in the perivascular adipose tissue adjacent to the injured arteries [22], and this correlation could be due to the activity of the extracellular histone H4 [23]. Likewise, inhibition of PADs abrogated NETosis and reduced both vascular infiltration and atherosclerotic burden in ApoE-knockout mice [24], and neutralization of histone H4 prevented vascular smooth muscle cell dysfunction and atherosclerotic lesion destabilisation [23].

Citrullinated histone H3 can induce endothelial dysfunction, a process underlying the early stages of atherosclerosis [25]. Similarly, NETs provoked an increased production of MMP-2 by endothelial cells suggestive of endothelial dysfunction [26]. Activated endothelial cells promoted NETosis in an interleukin-8-dependent manner and were additionally damaged by NETs expelled during their co-culture with neutrophils [27, 28]. These findings suggest that citrullinated proteins may be a prerequisite of plaque formation by inducing endothelial dysfunction, vascular smooth muscle cell activation, and extracellular matrix remodelling [29-33]. Yet, most of the studies focused on the role of NETs on sequelae of plaque development (*e.g.,* plaque erosion and rupture together resulting in atherothrombosis) and on the composition of NET-associated proteins within the thrombi, rather than on investigating citrullination within the atherosclerotic plaque. Hence, there is no direct evidence that could establish a pathophysiological paradigm linking citrullination with atherosclerosis development.

Here, we interrogated human atherosclerotic plaques for citrullination signatures. Immunostaining demonstrated that peptidylcitrulline and PADI2 are almost exclusively present in the atherosclerotic tissue but not in the intact tunica media. Citrullinated histone H3 and myeloperoxidase were also overrepresented in the neointima, although NETs have not been clearly visualised. Protein profiling also showed elevated levels of peptidylcitrulline, PADI2, and PADI4 in atherosclerotic plaques compared to the adjacent arterial segments. PADI2 and peptidylcitrulline were co-localised with the cells in the neointima; moreover, PADI2 was co-localised with an extracellular vesicle marker CD81 [34-39]. Serum levels of anti-citrullinated protein antibodies (ACPAs) did not differ in patients with ischaemic stroke and healthy volunteers indicating that citrullination is a local but not systemic phenomenon. Collectively, our results advocate the pathophysiological relevance of citrullination for the development of atherosclerosis and furnish a rationale for the more frequent use of non-specific anti-inflammatory therapies in the prevention of ischaemic events.

## 2 MATERIALS AND METHODS

This section contains Brief Materials and Methods applied to perform the experiments. Detailed Materials and Methods are provided in the Supplementary Files.

### 2.1 Patients and Samples

Carotid atherosclerotic plaques (n = 14) and adjacent arterial segments (n = 14) were pairwise excised during the carotid endarterectomy from 14 patients admitted to the Research Institute for Complex Issues of Cardiovascular Diseases (Kemerovo, Russia). The collection of clinical specimens was approved by the Local Ethical Committee of the Research Institute for Complex Issues of Cardiovascular Diseases (ethical approval code 25/2021, date of approval: 02 September 2021), and written informed consent has been provided by all study participants after receiving a full explanation of the study’s purposes. The investigation was carried out in accordance with the Good Clinical Practice and the latest revision of the Declaration of Helsinki (2013). Criteria of inclusion were: 1) performance of carotid endarterectomy due to chronic brain ischemia or ischemic stroke; 2) a signed written informed consent to be enrolled. A criterion of exclusion was incomplete investigation regardless of the reason; in this case, we enrolled another subject with similar age, gender and clinicopathological features who met the inclusion criteria. The clinicopathological features of the patients are presented in Table **1**. Detailed information on diagnostics of these features is indicated in Detailed Materials and Methods.

For serum measurements, we used the samples collected in our previous study (44 serum samples from asymptomatic blood donors and 44 serum samples from patients who underwent carotid endarterectomy because of ischaemic stroke [40] and the respective commercially available enzyme-linked immunosorbent assay (ELISA) kit which quantitates anti-citrullinated protein antibodies (ACPAs, KS-050, Omnix, Saint Petersburg, Russia). Colorimetric measurement of ACPAs was conducted according to the manufacturer’s protocol using a Multiskan Sky microplate spectrophotometer (Thermo Fisher Scientific, Waltham, MA, USA).

### 2.2 Isolation of RNA and Total Protein

Upon the excision, atherosclerotic plaques were cut into 3 ring segments. Isolation and quantification of RNA and total protein were isolated as previously described [40, 41]. Upon the excision, atherosclerotic plaques were cut into 3 ring segments. The second and third segments as well as the pieces of the adjacent arterial segment were homogenised in TRIzol Reagent (15596018, Thermo Fisher Scientific, Waltham, MA, USA) for RNA extraction or in T-PER Tissue Protein Extraction Reagent (78510, Thermo Fisher Scientific, Waltham, MA, USA) supplied with Halt protease and phosphatase inhibitor cocktail (78444, Thermo Fisher Scientific, Waltham, MA, USA) for the total protein extraction according to the manufacturer’s protocols. With regards to the total protein extraction, two rounds of sample enrichment have been performed: initial centrifugation at 14,000 × g (Microfuge 20R, Beckman Coulter, Brea, CA, USA) for 10 minutes and subsequent ultracentrifugation at 200,000 × g (Optima MAX-XP, Beckman Coulter, Brea, CA, USA) for 1 hour to sediment insoluble supramolecular complexes. Quantification and quality control of the isolated RNA was performed employing Qubit 4 fluorometer (Q33238, Thermo Fisher Scientific, Waltham, MA, USA) and respective consumables (Qubit RNA BR, Q10210; Qubit RNA IQ, Q33222; Qubit RNA IQ standards for calibration, Q33235; Qubit assay tubes, Q32856, Thermo Fisher Scientific, Waltham, MA, USA) according to the manufacturer’s protocols. Quantification of total protein was conducted using BCA Protein Assay Kit (23227, Thermo Fisher Scientific, Waltham, MA, USA) and Multiskan Sky microplate spectrophotometer (Thermo Fisher Scientific, Waltham, MA, USA) in accordance with the manufacturer’s protocol.

### 2.3 Proteomic Profiling

Sample preparation and shotgun proteomics analysis have been conducted as previously described [42-47]. Bioinformatic analysis was also conducted as previously described [48-61]. The mass spectrometry proteomics data have been deposited to the ProteomeXchange Consortium *via* the PRIDE [48] partner repository with the dataset identifier PXD035173.

Upon the removal of T-PER Buffer by acetone precipitation (650501, Sigma-Aldrich, Saint Louis, MO, USA), the protein pellet was resuspended in 8 mol/L urea (U5128, Sigma-Aldrich, Saint Louis, MO, USA) diluted in 50 mmol/L ammonium bicarbonate (09830, Sigma-Aldrich, Saint Louis, MO, USA). The protein concentration was measured by Qubit 4 fluorometer (Q33238, Thermo Fisher Scientific, Waltham, MA, USA) with QuDye Protein Quantification Kit (25102, Lumiprobe, Moscow, Russia) according to the manufacturer’s protocol. Protein samples (10 μg) were then incubated in 5 mmol/L dithiothreitol (D0632, Sigma-Aldrich, Saint Louis, MO, USA) for 1 hour at 37°C with the subsequent incubation in 15 mmol/L iodoacetamide for 30 minutes in the dark at room temperature (I1149, Sigma-Aldrich, Saint Louis, MO, USA). Next, the samples were supplied with 5 mmol/L dithiothreitol, immediately diluted with 7 volumes of 50 mmol/L ammonium bicarbonate and incubated for 16 hours at 37°C with 200 ng of trypsin (1:50 trypsin: protein ratio; VA9000, Promega, Madison, WI, USA). The peptides were then frozen at −80°C for 1 hour and desalted with stage tips (Tips-RPS-M.T2.200.96, Affinisep, Le Houlme, France), according to the manufacturer’s protocol using methanol (1880092500, Sigma-Aldrich, Saint Louis, MO, USA), acetonitrile (1000291000, Sigma-Aldrich, Saint Louis, MO, USA), and 0.1% formic acid (33015, Sigma-Aldrich, Saint Louis, MO, USA). Desalted peptides were dried in a centrifuge concentrator (Concentrator plus, Eppendorf, Hamburg, Germany) for 3 hours and finally dissolved in 20 µL 0.1% formic acid for further shotgun proteomics analysis.

Approximately 500 ng of peptides were used for shotgun proteomics analysis by ultra-high performance liquid chromatography-tandem mass spectrometry analysis with ion mobility (UHPLC-MS/MS) in TimsToF Pro mass spectrometer (Bruker Daltonics, Billerica, MA, USA) with nanoElute UHPLC system (Bruker Daltonics, Billerica, MA, USA). UHPLC was performed in the two-column separation mode with Acclaim PepMap 5 mm Trap Cartridge (Thermo Fisher Scientific, Waltham, MA, USA) and Bruker Ten separation column (C18 ReproSil AQ, 100 mm × 0.75 mm, 1.9 µm, 120 A; Bruker Daltonics, Billerica, MA, USA) in a gradient mode with 500 nL/min flow rate and 50°C. Phase A was water/0.1% formic acid, phase B was acetonitrile/0.1% formic acid. The gradient was from 2% to 30% phase B for 16 minutes, then to 38% phase B for 5 minutes and to 95% phase B for 3 minutes with subsequent washing with 95% phase B for 6 minutes. Before each sample, trap and separation columns were equilibrated with 10 and 4 column volumes, respectively. The captiveSpray ion source was used for electrospray ionization with 1600 V of capillary voltage, 3 L/min N_2_ flow, and 180°C source temperature.

The mass-spectrometry acquisition was performed in data-independent acquisition mode with trapped ion mobility spectroscopy (TIMS) in PASEF (parallel accumulation serial fragmentation) mode (diaPASEF) consisting of 5 cycles with 3 or 2 mobility windows each including a total of 13 mass width windows (50 Da width) covering the m/z range from 457.81 to 1046.80 and ion mobility range (1/K0) from 0.85 to 1.18 V s/cm^2^. The collision energy was programmed as a function of ion mobility, following a straight line from 20 eV for 1/K0 of 0.85V s/cm^2^ to 59 eV for 1/K0 of 1.3 V s/cm^2^.

Protein identification was performed in DIA-NN software (v. 1.8) using the human protein SwissProt database (https://www.uniprot.org/; accessed on 14 December 2021; organism: Human [960^6^]; uploaded on 2 March 2021; 20,394 sequences). Using the database, we performed library generation (library-free search) by the default DIA-NN settings. The generated library consisted of 20,338 proteins with 8,454,641 precursors. The search parameters were: parent mass and fragment mass error tolerance error 10 ppm, protein and peptide FDR < 1%, two possible missed cleavage sites, and proteins with ≥ 2 unique peptides. Cysteine carbamidomethylation was set as a fixed modification.

Label-free quantification by peak area under the curve was used for further analysis in R (version 3.6.1; R Core Team, 2019). All proteins presented in 12 of 14 biological replicates were identified and the groups were compared by the “VennDiagram” package [49] and drawing of the Venn diagram. The proteins with NA in > 2 samples were removed and the imputation of missed values by k-nearest neighbors was performed by the “impute” package [50]. Further, log-transformation and quantile normalization with further analysis of differential expression by the “limma” package [51] were performed. Finally, we performed clusterization of samples by partial least squares discriminant analysis in the “MixOmics” package [52]. “ggplot2” [53] and “pheatmap” [54] packages were used for visualisation. Reproducible code for data analysis is available from https://github.com/ArseniyLobov/Proteomic-profiling-of-atherosclerotic-plaques (accessed on 31 July 2022). Differentially expressed proteins were defined as those with logarithmic fold change ≥ 1 and false discovery rate-corrected *p* value ≤ 0.05. Bioinformatic analysis was performed using Gene Ontology [55, 56], Reactome [57, 58], UniProtKB Keywords [59], and Kyoto encyclopaedia of genes and genomes (KEGG) databases [60, 61].

### 2.4 Measurement of Gene and Protein Expression

Gene expression (*CD9*, *CD63*, *CD81*, *PADI2,* and *PADI4* genes) was measured as previously described [40, 62-64]. RNA quantification was conducted as described above and was followed by a reverse transcription using a High Capacity cDNA Reverse Transcription Kit (4368814, Thermo Fisher Scientific, Waltham, MA, USA) and quantitative polymerase chain reaction (RT-qPCR) using customised primers (500 nmol/L each, Evrogen, Moscow, Russia), cDNA (20 ng), and PowerUp SYBR Green Master Mix (A25778, Thermo Fisher Scientific, Waltham, MA, USA) according to the manufacturers’ protocols. Primer sequences are provided in Table **2**. Technical replicates (n = 3 per sample) were performed in all RT-qPCR experiments. The reaction was considered successful if its efficiency was 90-105% and R^2^ was ≥ 0.98. Quantification of *CD9*, *CD63*, *CD81*, *PADI2* and *PADI4* mRNA levels in carotid atherosclerotic plaques (n = 8) and adjacent intact arterial segments (n = 8) was performed by using the 2^−ΔΔCt^ method. Relative transcript levels were expressed as a value relative to the average of 3 housekeeping genes (*ACTB*, *GAPDH*, *B2M*). A fresh sample of the human liver obtained from the patient who underwent liver transplantation was used as a positive control.

For measuring protein expression in carotid atherosclerotic plaques (n = 12) and adjacent arterial segments (n = 12), protein quantification, protein loading (15 μg per sample), separation, and transfer were performed as in [40, 41, 46]. Equal amounts of protein (15 μg per sample) were mixed with NuPAGE lithium dodecyl sulfate sample buffer (NP0007, Thermo Fisher Scientific, Waltham, MA, USA) at a 4:1 ratio and NuPAGE sample reducing agent (NP0009, Thermo Fisher Scientific, Waltham, MA, USA) at a 10:1 ratio, denatured at 99°C for 5 minutes and then loaded on a 1.5 mm NuPAGE 4–12% Bis-Tris protein gel (NP0335BOX, Thermo Fisher Scientific, Waltham, MA, USA). The 1:1 mixture of Novex Sharp pre-stained protein standard (LC5800, Thermo Fisher Scientific, Waltham, MA, USA) and MagicMark XP Western protein standard (LC5602, Thermo Fisher Scientific, Waltham, MA, USA) was loaded as a molecular weight marker. Proteins were separated by the sodium dodecyl sulphate-polyacrylamide gel electrophoresis (SDS-PAGE) at 150 V for 2 hours using NuPAGE 2-(N-morpholino)ethanesulfonic acid SDS running buffer (NP0002, Thermo Fisher Scientific, Waltham, MA, USA), NuPAGE Antioxidant (NP0005, Thermo Fisher Scientific, Waltham, MA, USA), and XCell SureLock Mini-Cell vertical mini-protein gel electrophoresis system (EI0001, Thermo Fisher Scientific, Waltham, MA, USA). Protein transfer was performed using polyvinylidene difluoride (PVDF) transfer stacks (IB24001, Invitrogen) and iBlot 2 Gel Transfer Device (Invitrogen) according to the manufacturer’s protocols using a standard transfer mode for 30–150 kDa proteins (P0—20 V for 1 minute, 23 V for 4 minutes, and 25 V for 2 minutes). PVDF membranes were then incubated in iBind Flex Solution (SLF2020, Solution Kit Thermo Fisher Scientific, Waltham, MA, USA) for 1 hour to prevent non-specific binding.

Blots were probed with rabbit antibodies to PADI2 (1:500, ab183194, Abcam, Cambridge, UK) or mouse antibodies to PADI4 (1:500, H00023569-M01, Novus Biologicals, Centennial, CO, USA), peptidylcitrulline (1:500, MABN328, Sigma-Aldrich, Saint Louis, MO, USA), and glyceraldehyde-3-phosphate dehydrogenase (GAPDH, loading control, 1:250, ab139416, Abcam, Cambridge, UK). Horseradish-peroxidase-conjugated goat anti-rabbit (7074, Cell Signaling Technology, Danvers, MA, USA) or goat anti-mouse secondary antibodies (AP130P, Sigma-Aldrich, Saint Louis, MO, USA) were used at 1:200 and 1:1000 dilution, respectively. Incubation with the antibodies was performed using iBind Flex Solution Kit (SLF2020, Thermo Fisher Scientific, Waltham, MA, USA), iBind Flex Cards (SLF2010, Thermo Fisher Scientific, Waltham, MA, USA) and iBind Flex Western Device (SLF2000, Thermo Fisher Scientific, Waltham, MA, USA) during 3 h according to the manufacturer’s protocols. Chemiluminescent detection was performed using SuperSignal West Pico PLUS chemiluminescent substrate (34580, Thermo Fisher Scientific, Waltham, MA, USA) and C-DiGit blot scanner (LI-COR Biosciences, Linkoln, NE, USA) in a high-sensitivity mode (12-min scanning). To properly assess the protein loading, we conducted total protein normalization using 0.1% Fast Green FCF, a protein-specific dye (F8130, Solarbio Life Sciences, Beijing, China), and Odyssey XF imaging system (LI-COR Biosciences, Lincoln, NE, USA) in a 600 nm channel (520 nm excitation and 600 nm emission). Densitometry was performed as in [65] using the ImageJ software (National Institutes of Health, Bethesda, MD, USA) using the standard algorithm (consecutive selection and plotting of the lanes with the measurement of the peak area) and subsequent adjustment to the total protein loading in the respective lanes.

### 2.5 H&E Staining and Immunostaining

The ring segments of atherosclerotic plaques were snap-frozen in the optimal cutting temperature compound (Tissue-Tek, 4583, Sakura Finetek, Tokyo, Japan) using liquid nitrogen and were then sectioned on a cryostat (Microm HM 525, Thermo Scientific, Waltham, MA, USA) as in our previous studies [40, 66]. To ensure the proper immunofluorescence examination, we prepared 8 sections (7 µm thickness), evenly distributed across the entire carotid artery segment, per slide. Sections were then stained with haematoxylin and eosin (ab245880, Abcam, Cambridge, UK) according to the manufacturer’s protocol and visualised by light microscopy (AxioImager.A1 microscope and EC Plan-Neofluar 20×/0.50 or EC Plan-Neofluar 40×/0.75 M27 objectives, Carl Zeiss, Oberkochen, Germany). For the immunofluorescence staining, the sections were fixed in ice-cold acetone (6-09-20-03-83, EKOS-1, Moscow, Russia) for 10 minutes and blocked in 1% bovine serum albumin (Р091Е, PanEco, Moscow, Russia) for 1 hour to prevent non-specific binding. Sections were then incubated for 16 hours at 4°C with the following antibodies:

1. Mouse antibodies to peptidylcitrulline (1:250, MABN328, Sigma-Aldrich, Saint Louis, MO, USA) in combination with rabbit antibodies to PADI2 (1:100, ab183194, Abcam, Cambridge, UK);
2. Mouse antibodies to peptidylcitrulline (1:250, MABN328, Sigma-Aldrich, Saint Louis, MO, USA) in combination with rabbit antibodies to CD9 (1:50, NBP2-67310, Novus Biologicals, Centennial, CO, USA);
3. Rabbit antibodies to PADI2 (1:100, ab183194, Abcam, Cambridge, UK) in combination with mouse antibodies to CD81 (1:100, NBP1-44861, Novus Biologicals, Centennial, CO, USA);
4. Rabbit antibodies to citrullinated histone H3 (1:500, NB100-57135; Novus Biologicals, Centennial, CO, USA) in combination with mouse antibodies to NE/ELA2 (1:50, MAB91671-100, R&D Systems, Minneapolis, MN, USA);
5. Rabbit antibodies to MPO (1:100, ab208670, Abcam, Cambridge, UK) as a single stain.

The next day, sections were further treated with donkey anti-rabbit or anti-mouse pre-adsorbed Alexa Fluor-488-conjugated (1:500, ab150061 or ab150109, Abcam, Cam-bridge, UK) and donkey anti-rabbit or anti-mouse pre-adsorbed Alexa Fluor-555-conjugated (1:500, ab150062 or ab150110, Abcam, Cambridge, UK) secondary antibodies for 1 hour at room temperature. Nuclei were counterstained with 4′,6-diamidino-2-phenylindole (DAPI) (10 μg/mL, D9542, Sigma-Aldrich, Saint Louis, MO, USA) for 30 minutes at room temperature. Between all steps excluding blocking of non-specific binding, washing was performed thrice with 0.1% phosphate-buffered saline (60201, Pushchino Laboratories, Pushchino, Russia) solution of Tween-20 (P9416, Sigma-Aldrich, Saint Louis, MO, USA). Coverslips were mounted with ProLong Gold Antifade (P36934, Thermo Fisher Scientific, Waltham, MA, USA). Slides were examined by confocal laser scanning microscopy (LSM 700, Carl Zeiss, Oberkochen, Germany). Semi-quantitative analysis of signal intensity in confocal images and co-localisation analysis were performed using the respective ImageJ (National Institutes of Health, Bethesda, MD, USA) plugins (Colour Histogram, Colocalisation Threshold and Coloc2) upon the correction for the background signal obtained from the negative control images. To evaluate the co-localisation, we calculated Pearson's r above threshold (zero-zero pixels), thresholded Mander's split co-localisation coefficient (the proportion of signal in each channel that co-localizes with the other channel) for both (red and green) channels, percent volume co-localised with each channel, and intensity volume above threshold co-localised with each channel in both neointima and media.

### 2.6 Statistical Analysis

Statistical analysis was performed using GraphPad Prism 8 (GraphPad Software, San Diego, CA, USA). For descriptive statistics, data were presented as median, 25^th^ and 75^th^ percentiles (interquartile range), and range (the distance between minimum and maximum values). Two independent groups were compared by the Mann–Whitney U-test. The *p* values ≤ 0.05 were regarded as statistically significant.

## 3 RESULTS

**Fig. (1):**
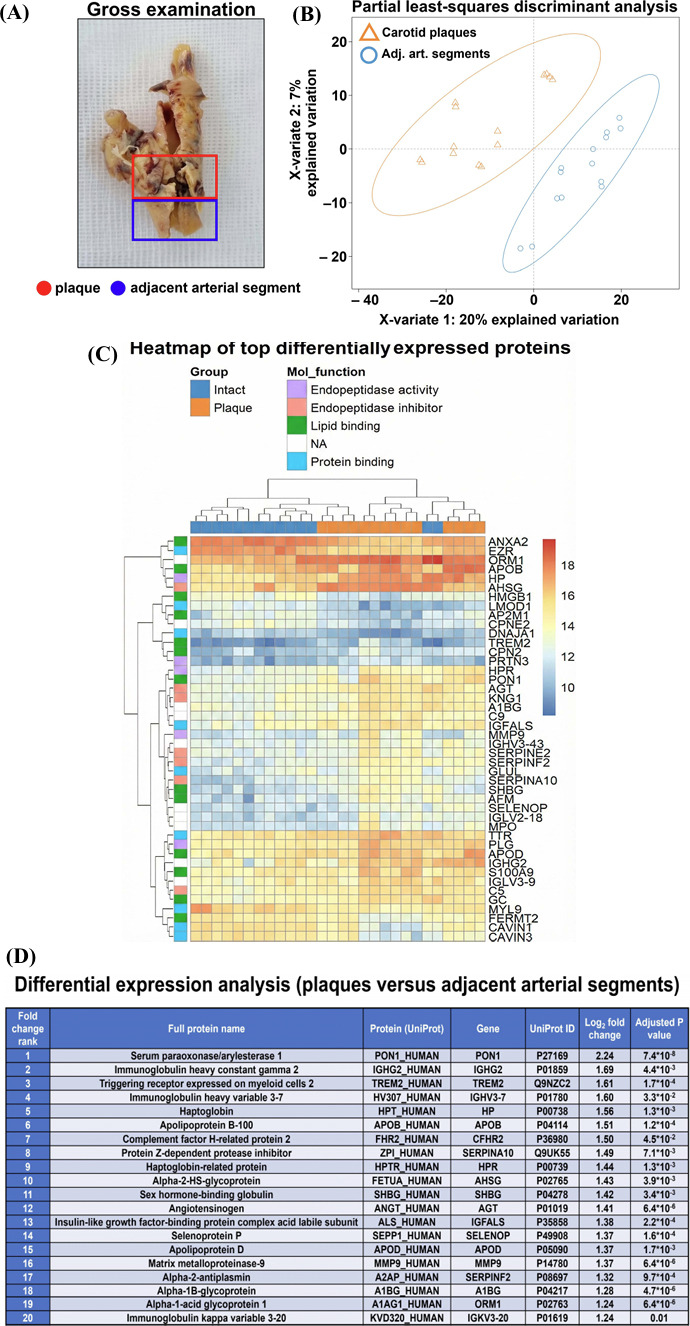
Ultra-high performance liquid chromatography-tandem mass spectrometry analysis of 14 atherosclerotic plaques and adjacent arterial segments collected during the carotid endarterectomy. (**A**) Gross examination of the specimen. Plaque and adjacent arterial segment are demarcated by red and blue contour, respectively; (**B**) Partial-least squares discriminant analysis demonstrates two separate clusters delineating the significant differences between plaques and adjacent arterial segments; (**C**) Heatmap of top differentially expressed proteins in plaques (orange) and intact tissue (*i.e.,* adjacent arterial segments) confirming clear clusterisation of plaque and near-plaque proteomic profiles; (**D**) Among the top 20 proteins overexpressed in plaques are catalytic lipid-associated bioscavenger paraoxonase 1, atherogenic apolipoprotein B-100, iron-associated protein haptoglobin, and matrix metalloproteinase-9 all indicative of advanced atherosclerosis.

To perform an objective analysis of protein citrullination in atherosclerotic plaques, we collected 14 atherosclerotic plaques and adjacent arterial segments excised during carotid endarterectomy (Fig. **1A**) and profiled them using ultra-high performance liquid chromatography-tandem mass spectrometry analysis (UHPLC-MS/MS). Partial least-squares discriminant analysis (Fig. **1B**) and dendrogram (Fig. **1C**) indicated clusterisation of the samples, as 213 plaque-specific and 111 intact arteria-specific proteins have been identified. Further, 46 proteins were upregulated (fold change ≥ 2 and FDR-adjusted *p* value ≤ 0.05) and 13 proteins were downregulated (fold change ≤ 0.5 and FDR-adjusted *p* value ≤ 0.05) in plaques in comparison with the adjacent arterial segments. The top 20 proteins overexpressed in plaques included catalytic lipid-associated bioscavenger paraoxonase 1, atherogenic apolipoprotein B-100, iron-associated protein haptoglobin, and matrix metalloproteinase-9 (Fig. **1D**). Taken together, these findings demonstrated the different molecular portfolios of plaques and adjacent arterial segments included in further examination, indicating the advanced stage of plaque progression.

Bioinformatics analysis of proteins which were classified as specific or upregulated in plaques and adjacent arterial segments revealed that both of these sample groups showed active immune response and particularly response to bacteria manifested as complement activation (complement activation: 10 DEPs in plaques and 11 DEPs in the intact arterial segments, fold change: 5.85 and 13.36, respectively; defense response to bacterium: 14 DEPs in plaques and 14 DEPs in the intact arterial segments, fold change: 3.12 and 6.47, respectively; regulation of complement cascade: 13 DEPs in plaques and 10 DEPs in the intact arterial segments, fold change: 9.62 and 15.34, respectively; initial triggering of complement: 10 DEPs in plaques and 6 DEPs in the intact arterial segments, fold change: 9.57 and 11.91, respectively; complement cascade: 14 DEPs in plaques and 10 DEPs in the intact arterial segments, fold change: 9.49 and 14.06, respectively), phagocytosis (phagocytosis, engulfment: 10 DEPs in plaques and 12 DEPs in the intact arterial segments, fold change: 6.21 and 15.46, respectively; phagocytosis, recognition: 8 DEPs in plaques and 11 DEPs in the intact arterial segments, fold change: 6.14 and 17.51, respectively; phagocytosis: 15 DEPs in plaques and 15 DEPs in the intact arterial segments, fold change: 3.08 and 1.49, respectively, endocytosis: 21 DEPs in plaques and 15 DEPs in the intact arterial segments, fold change: 3.29 and 4.88, respectively, role of phospholipids in phagocytosis: 11 DEPs in plaques and 6 DEPs in the intact arterial segments; fold change: 10.17 and 11.51, respectively), and neutrophil activation (neutrophil degranulation: 19 DEPs in plaques and 15 DEPs in the intact arterial segments, fold change: 3.24 and 5.31, respectively) (Tables **S1**, **S2**). Both tissue types also showed protein signatures of active extracellular vesicle production (vesicle-mediated transport: 43 DEPs in plaques and 23 DEPs in the intact arterial segments, fold change: 2.60 and 2.88, respectively; secretory granule lumen: 20 DEPs in plaques and 12 DEPs in the intact arterial segments, fold change: 5.09 and 6.33, respectively; extracellular vesicle: 76 DEPs in plaques and 39 DEPs in the intact arterial segments, fold change: 2.91 and 3.10, respectively; secretory granule: 30 DEPs in plaques and 18 DEPs in the intact arterial segments, fold change: 2.79 and 3.47, respectively, secretory vesicle: 32 DEPs in plaques and 18 DEPs in the intact arterial segments, fold change: 2.50 and 2.91, respectively) (Tables **S1**, **S3**). Plaques were notable for the abundance of molecular categories related to innate immune response (activation of innate immune response: 8 DEPs, fold change: 8.24; acute inflammatory response: 8 DEPs, fold change: 8.04; inflammatory response: 19 DEPs, fold change: 2.93), oxidative stress (respiratory burst involved in defense response: 4 DEPs, fold change: 40.69; respiratory burst: 4 DEPs, fold change: 13.02; response to oxygen-containing compound: 39 DEPs, fold change: 2.08; regulation of TLR by endogenous ligand: 4 DEPs, fold change: 15.50; interferon signaling: 9 DEPs, fold change: 3.72; NF-κB binding: 5 DEPs, fold change: 12.72), biosynthetic processes including translation and transcription (translation: 17 DEPs, fold change: 3.67; peptide biosynthetic process: 17 DEPs, fold change: 3.42; amide biosynthetic process: 18 DEPs, fold change: 2.82; cellular macromolecule biosynthetic process: 23 DEPs, fold change: 2.45; regulation of cellular component biogenesis: 28 DEPs, fold change: 2.38; eukaryotic translation initiation: 7 DEPs, fold change: 4.79; eukaryotic translation elongation: 6 DEPs, fold change: 5.19; eukaryotic translation termination: 6 DEPs, fold change: 5.25; translation regulator activity: 9 DEPs, fold change: 4.92; transcription coactivator activity: 13 DEPs, fold change: 3.76), coagulation (regulation of coagulation: 6 DEPs, fold change: 6.60; platelet degranulation: 10 DEPs, fold change: 6.41; platelet activation, signaling and aggregation: 12 DEPs, fold change: 3.76) and regulated cell death (positive regulation of cell death: 20 DEPs, fold change: 2.81) (Tables **S1**, **S2**, and **S4**). In contrast, adjacent arterial segments displayed fingerprints of B cell activation (positive regulation of B cell activation: 10 DEPs, fold change: 9.93, immunoglobulin production: 7 DEPs, fold change: 7.38) and adaptive immune response (lymphocyte mediated immunity: 13 DEPs, fold change: 8.85; positive regulation of lymphocyte activation: 13 DEPs, fold change: 5.35; adaptive immune response: 19 DEPs, fold change: 4.92) (Table **S1**).

**Fig. (2):**
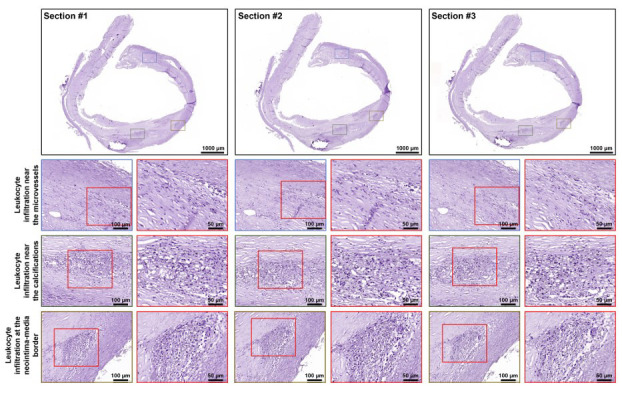
Staining of atherosclerotic plaques with haematoxylin and eosin shows the leukocyte infiltrations near the microvessels (top), calcifications (middle), and at the neointima-media border (bottom). Overview images: magnification: ×1.6, scale bar: 1000 µm; close-ups demarcated by blue, green, and gold contour: magnification: ×200, scale bar: 100 µm; close-ups demarcated by red contour: magnification: ×400, scale bar: 50 µm.

To assess the amounts and localisation of NETs and leukocyte infiltrations, we conducted haematoxylin and eosin staining of 12 plaques (3 consecutive sections per each). We have not found uncondensed chromatin in any of the samples regardless of the section (Fig. **2**) but have revealed severe leukocyte infiltration near the microvessels, calcifications, and at the border between neointima and tunica media (Fig. **2**). Within the neointima, we have detected microvessels variable in size and shape (Fig. **S2**). Calcifications were observed at the border between neointima and tunica media and have been often located in close vicinity to leukocyte infiltrations (Fig. **S3**). Leukocyte amount near blood vessels and calcifications (Fig. **S4A**) was lower than at the border between the intima and tunica media (Fig. **S4B**) where leukocytes could coalesce into clusters (Fig. **S4C**).

**Fig. (3):**
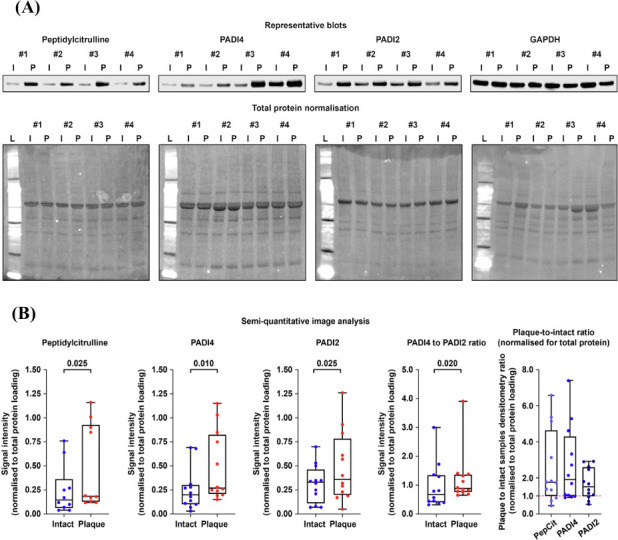
Western blotting profiling of carotid atherosclerotic plaques and adjacent arterial segments. (**A**) Representative Western blots of plaques (P) and adjacent arterial segments (I) probed for peptidylcitrulline (PepCit), PADI4, PADI2, or GAPDH (top), loading control was performed by GAPDH blotting and total protein normalisation (bottom); (**B**) Semi-quantitative image analysis of abovementioned Western blots by means of densitometry (with respect to the total protein normalisation); the results are provided after the adjustment of specific signal for the total protein loading. Blue and red dots indicate intact arterial segments and plaques, respectively. Each dot on the plots represents the measurement from one sample (n = 12 samples per group). Whiskers indicate the range, box bounds indicate the 25^th^–75^th^ percentiles, and centre lines indicate the median. *p* values are provided above boxes, Mann–Whitney U test.

We next asked whether carotid plaques are enriched with peptidylcitrulline, PADI2, and PADI4 as compared with adjacent arterial segments. Western blotting measurements performed in 12 pairwise collected plaques and intact arteries found a statistically significant increase in peptidylcitrulline, PADI4 and PADI2 in plaques, and PADI4 prevailed over PADI2 as indicated by elevated PADI4 to PADI2 ratio (Figs. **3A** and **B**). However, plaques were devoid of *PADI2* mRNA and *PADI4* mRNA was also expressed in negligible amounts, although *PADI4* was highly expressed in the liver (Table **3**). Genes encoding extracellular vesicle markers (*CD9*, *CD63*, and *CD81*) have shown notable expression in plaque lysate, confirming the technical validity of the assay (Table **3**). Hence, PADI2 and PADI4 might be delivered to the carotid plaques *via* systemic circulation.

**Fig. (4):**
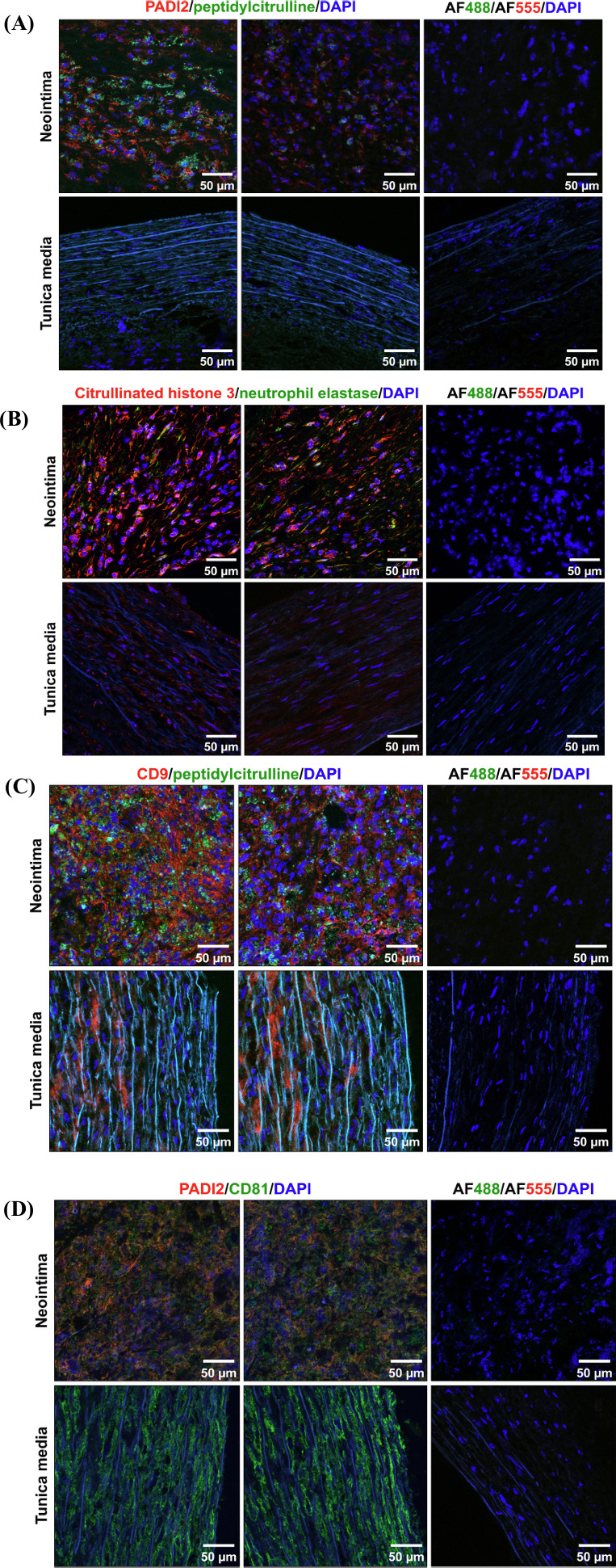
Immunofluorescence staining of neointima and adjacent tunica media for: (**A**) PADI2 (red colour) and peptidylcitrulline (green colour); (**B**) citrullinated histone H3 (red colour) and neutrophil elastase (green colour); (**C**) CD9 (red colour) and peptidylcitrulline (green colour); (**D**) PADI2 (red colour) and CD81 (green colour). Nuclei are counterstained with DAPI (blue colour). Two representative images per staining are provided. Negative controls (*i.e.,* sections stained with species-specific fluorescent-labelled secondary antibodies and DAPI but without the respective antigen-specific primary antibodies) are provided to the right from representative images. Magnification: ×400, scale bar: 50 µm. Note that PADI2, peptidylcitrulline, and citrullinated histone H3 are abundant in the neointima but absent or downregulated in the tunica media. In contrast to peptidylcitrulline, a large proportion of PADI2 is co-localised with CD81-positive cells or extracellular vesicles (yellow colour formed by co-localisation of red and green colours).

To verify these results, we focused on plaque segments which contained neointima and a few layers of intact tunica media and stained them for PADI2, peptidylcitrulline, citrullinated histone H3, NE/ELA2, and extracellular vesicle markers CD9 and CD81. Strikingly, both PADI2 and peptidylcitrulline were exclusively detected in the neointima but not tunica media (Fig. **4A**) and citrullinated histone H3 was considerably upregulated in the neointima, although it was also observed in tunica media (Fig. **4B**, **S5**, **S6**). Citrullinated histone H3 was not associated with NE/ELA2 and shapeless, uncondensed chromatin (a feature of NETs) has not been revealed (Fig. **4B**, **S5**, **S6**). Peptidylcitrulline has not been associated with the extracellular vesicles (Fig. **4C**) whilst PADI2 was largely co-localised with CD81 in the neointima (Fig. **4D**). In accordance with the aforementioned findings, CD81-positive regions in the tunica media were devoid of PADI2 (Fig. **4D**).

**Fig. (5):**
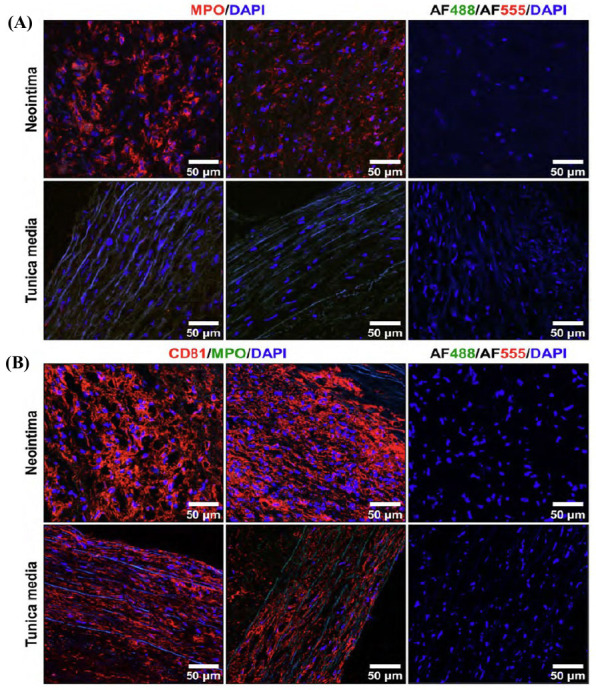
Immunofluorescence staining of neointima and adjacent tunica media for (**A**) neutrophil and macrophage marker MPO (red colour); (**B**) MPO and CD81, a tetraspanin enriched in extracellular vesicles but also expressed on cell membranes. Nuclei are counterstained with DAPI (blue colour). Two representative images per staining are provided. Negative controls (*i.e.,* sections stained with species-specific fluorescent-labelled secondary antibodies and DAPI but without antigen-specific antibody to MPO) are provided to the right from representative images. Magnification: ×400, scale bar: 50 µm. Note that MPO is significantly upregulated in the neointima as compared with the tunica media.

Albeit some of the plaque regions contained considerable amounts of neutrophil and macrophage marker MPO in the neointima but not in tunica media (Fig. **5A**), there has been no colocalisation of MPO and CD81, which was abundantly expressed in the medial layer (Fig. **5B**, **S7**). However, we did not find any complexes of unwound chromatin which could indicate NETs, in agreement with the findings from haematoxylin and eosin staining.

**Fig. (6):**
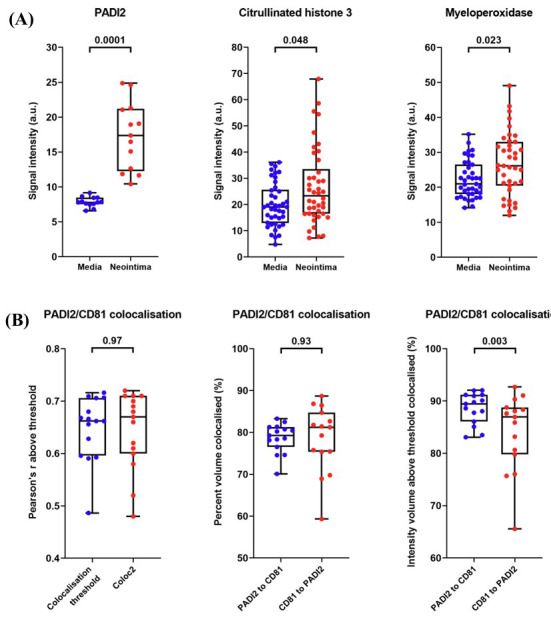
Semi-quantitative analysis of immunofluorescence staining of neointima and adjacent tunica media. (**A**) Comparison of signal intensity (measured in ImageJ in arbitrary units) by using the Colour Histogram plugin, blue and red dots indicate tunica media and neointima, respectively. (**B**) Analysis of PADI2 and CD81 colocalisation (ImageJ) by Colocalisation Threshold and Coloc2 plugins. Each dot on the plots represents the measurement from one image. Whiskers indicate the range, box bounds indicate the 25^th^–75^th^ percentiles, and centre lines indicate the median. *p* values are provided above boxes, Mann–Whitney U test.

Semi-quantitative analysis of signal intensity and co-localisation analysis of confocal images found statistically significant overexpression of PADI2, citrullinated histone H3, and myeloperoxidase in the neointima as compared to tunica media (Fig. **6A**). Irrespective of the tool for co-localisation assessment, from 70 to 90% of PADI2 was co-localised with CD81, a tetraspanin enriched in extracellular vesicles, suggesting a mechanism by which cells can regulate citrullination in the pathological environment (Fig. **6B**).

**Fig. (7):**
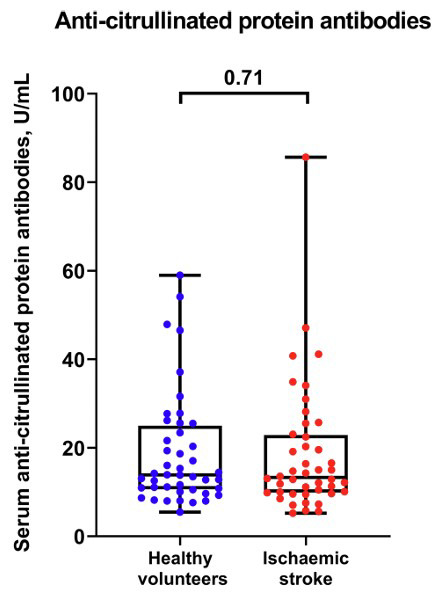
Enzyme-linked immunosorbent assay of serum ACPAs in healthy volunteers and patients with ischaemic stroke. Each dot on the plot represents the measurement from one subject. Whiskers indicate the range, box bounds indicate the 25^th^–75^th^ percentiles, and centre lines indicate the median. *p* value is provided above boxes, Mann–Whitney U test.

Finally, we measured the level of ACPAs in the serum from asymptomatic blood donors (n = 44) and patients with ischaemic stroke (n = 44). Although protein citrullination was evident in carotid plaques, serum ACPAs have not been augmented (Fig. **7**), assuming citrullination as a local but not systemic phenomenon in patients with carotid atherosclerosis.

## 4 DISCUSSION

Although it is estimated that human citrullinome encompasses as many as 250-300 proteins [67-75], the effects of citrullination in many organs and tissues are still poorly understood. Citrullination of histones plays an important role in the regulation of apoptosis [76] and differentiation [77] and is pivotal for the generation of NETs, complexes of decondensed citrullinated chromatin that are essential in the struggle with bacterial pathogens [78-80]. A number of the extracellular matrix proteins such as collagen [81-83], fibronectin [84-86] and fibrinogen [87-89], as well as cytoskeletal proteins tubulin [90, 91], vimentin [92, 93], and actin [94, 95] can also undergo citrullination.

Citrullination is among the primary mechanisms of autoimmune diseases (*e.g.,* rheumatoid arthritis, systemic lupus erythematosus, and multiple sclerosis) because the most important functional consequence of arginine-to-citrulline conversion is neoepitope formation [3-5, 96]. The pathophysiological importance of citrullination in rheumatoid arthritis is persuasively shown by ACPAs which are the gold standard for its diagnostics with a sensitivity from 60% (at the early stages) to 75% (at the late stages) and specificity > 90% [97, 98]. Immunostaining approaches demonstrated a higher prevalence of citrullinated proteins in the synovial sheath of patients with rheumatoid arthritis and muscles of patients with polymyositis in comparison with the corresponding healthy tissues [99]. Citrullinated proteins were also frequent in β-cells of pancreatic islets of diabetes-prone NOD mice [100] and areas of ongoing demyelination in patients with multiple sclerosis [101]. Taken together, these findings proposed that citrullination is an inflammation-associated rather than disease-associated protein modification. In keeping with this hypothesis, non-specific PAD inhibitor chloramidine suppressed inflammation by triggering apoptosis of immune cells in mice with dextran sulfate sodium-induced colitis [102]. Moreover, murine collagen-induced arthritis was ameliorated by either chloramidine [103] or specific PADI4 inhibitor GSK199 [104]. Notably, inflammation also potentiates citrullination, as pro-inflammatory cytokines interleukin-1β and γ-interferon induced citrullination in rat pancreas [100] while non-selective anti-inflammatory drugs (*e.g.,* glucocorticoids) reduced PADI4 expression and the amount of citrullinated proteins in joints of patients with rheumatoid arthritis [105]. Such interconnection between citrullination and aseptic inflammation points out the role of the former in cardiovascular pathology largely driven by both innate and adaptive immunity [2, 31, 32, 106-111].

Epidemiological studies have demonstrated that patients with rheumatoid arthritis have an elevated risk of subclinical atherosclerosis [112], major adverse cardiovascular events (*i.e.,* coronary artery disease and ischaemic stroke) [113-115], and cardiovascular death [116-118]. Notably, ACPAs are 3-fold more frequently detected in the serum of patients without rheumatoid arthritis but with coronary artery disease than in asymptomatic blood donors [119]. The presence of ACPAs in the serum of patients with myocardial infarction (also not suffering from rheumatoid arthritis) has been associated with a higher risk of cardiovascular death after 10 years of follow-up [120]. Likewise, high levels of ACPAs in patients with rheumatoid arthritis correlated with coronary artery calcification [121] and aortic calcification [18]. These epidemiological links indicated the clinical relevance of citrullination for cardiovascular disease. However, whether and how citrullination affects the development of atherosclerosis remains unclear. Proteomic studies found that carotid and aortic plaques isolated from patients without rheumatoid arthritis nonetheless have higher levels of citrullinated fibrinogen [18]. Subsequent immunohistochemical analysis of coronary plaques from a similar patient cohort revealed co-localisation of citrullinated proteins (including fibrinogen) with PADI4 [18].

In addition to the role of citrullination in the generation of neoepitopes in plaques, atherosclerosis is believed to be enhanced by the assemble of NETs which represent the supramolecular complexes composed of extruded, decondensed citrullinated chromatin which are released from neutrophils into the circulation and the extracellular matrix [30, 33, 122]. NETs also promote the development of inflammation [123-125] and clotting in injured [33] and healthy arteries [126]. Transplantation of PADI4-deficient bone marrow-derived cells to wild-type γ-irradiated mice, as well as intravenous administration of DNAse, prevented neointimal hyperplasia and thrombosis after electrical current-induced and cholesterol-rich diet-promoted endothelial injury [122]. Likewise, DNase-deficient mice were prone to pulmonary thrombosis as compared with wild-type animals [126].

In this study, we found that, in contrast to adjacent arterial segments, the proteome of carotid atherosclerotic plaques contains molecular signatures of activated innate immune response, response to lipopolysaccharide, NF-κB binding, endogenous TLR regulation, interferon signalling, respiratory burst, response to oxygen- containing compounds (potentially including reactive oxygen species), lysosomal transport, regulation of phagocytosis, positive regulation of cell death, organisation of extracellular vesicles, clathrin-coated vesicle cargo loading, and vesicle budding from the membrane. Taken together, these molecular categories suggested the involvement of sterile inflammation in the pathogenesis of atherosclerosis. Among the processes engaged in the development of aseptic inflammation, we focused on citrullination as its extent highly depends on innate immunity, oxidative stress, cell death, and phagocytosis [127-129] that collectively indicates citrullination as an inflammatory-dependent condition. Further, citrullination potentiates inflammation through generated neoepitopes as discussed above [130-133]. Therefore, we suggested that citrullination might be associated with the development of atherosclerotic plaques (*i.e.,* neointimal hyperplasia) and not be restricted to atherothrombosis occurring because of plaque erosion or rupture. As the proteomic analysis pointed at the possible involvement of extracellular vesicles in plaque progression, we have also interrogated the co-localisation of citrullination-specific enzymes with proteins which are enriched in extracellular vesicles. Unfortunately, we did not assess gut and thrombus microbiota composition and therefore were unable to evaluate its impact on plaque citrullination. We also did not study the impact of anti-ischemic therapy on the citrullination of histones or other citrullinated proteins, and fulfilling this task would require a higher sample size and a standardized technique for an objective assessment of protein citrullination within the plaque. These two shortcomings should be mentioned as the limitations of our study.

Further studies might investigate the efficacy of small molecule inhibitors (AFM-30a for PAD2; JBI-589, GSK199 and GSK484 for PAD4) in the prevention or treatment of cardiovascular disease. Application of these drugs provided successful inhibition of PAD2 and PAD4 in peripheral blood mononuclear cells [134] and neutrophils [134-137], in cell-free synovial fluid samples withdrawn from patients with rheumatoid arthritis [134], and in murine collagen-induced arthritis model [104, 137, 138]. Selective inhibitors of PAD4 (JBI-589 and GSK484) have prevented the development of inflammation-associated heart failure in this animal model by inhibiting neutrophil infiltration and halting the formation of neutrophil extracellular traps in the heart [138, 139]. Further, GSK484 has reduced the deposition of neutrophil extracellular traps and preserved endothelial integrity at sites of electrical current-induced and constrictive cuff-promoted intimal injury [140] and inhibited neutrophil extracellular trap-related thrombosis in mice [141]. GSK484 has also shown effectiveness in ameliorating giant cell myocarditis in rats [142]. It would also be feasible to test the aforementioned PAD inhibitors in combination with drugs that have previously shown anti-inflammatory effects in atherosclerotic plaque, such as sodium-glucose co-transporter-2 inhibitors and proprotein convertase subtilisin/kexin type 9 inhibitors [143-145]. Further, the effectiveness of PAD inhibitors might be also affected by the gut microbiota composition, which has been previously shown as a factor defining the severity of atherothrombosis in hyperglycemic patients with ST segment elevation myocardial infarction [146]. Collectively, these findings indicate the relevance of inhibiting PAD2 and PAD4 for the prevention and treatment of cardiovascular disease and underline the importance of respective pre-clinical and clinical trials in various settings. In particular, the abovementioned small molecule inhibitors of PAD2 (AFM-30a) and PAD4 (JBI-589, GSK199 and GSK484) hold promise for the prevention of endothelial injury and inflammation, two major pathological processes driving atherosclerosis.

## CONCLUSION

We found that citrullination frequently accompanies the development of carotid atherosclerotic plaques. Citrullinated proteins (*i.e.,* those distinguished by the antibodies to peptidylcitrulline), as well as PADI2, were detected in the neointima but not in the tunica media, suggesting that citrullination is largely specific for atherosclerosis and is rarely encountered in the intact blood vessels. However, peptidylcitrulline and PADI2/4 were also found in the arterial segments adjacent to the plaque, although in lower quantities than in established plaques. PADI2 and peptidylcitrulline were co-localised with the cells in the neointima, and 70–90% of PADI2 were co-localised with either neointimal cells or the extracellular vesicles (as CD81 is expressed both on cell and extracellular vesicle membranes). We speculate that citrullination (*i.e.,* the emergence of neoepitopes) might occur at any stage of atherosclerosis but does not involve tunica media even at severe stenosis or plaque rupture. However, we detected neither decondensed chromatin nor sporadic complexes of NE/ELA2, citrullinated proteins, and unwound chromatin signifying NETs. In addition, we have not documented any significant increase in ACPAs in the serum of patients with ischaemic stroke in comparison with healthy volunteers, defining citrullination as a local phenomenon in patients with established carotid plaques. Collectively, our findings support the pathophysiological link between the formation of neoepitopes and neointimal hyperplasia and provide another justification for the use of anti-inflammatory drugs (*e.g.,* non-steroidal anti-inflammatory drugs, selective cytokine inhibitors and small molecule PAD inhibitors) in the prevention of major adverse cardiovascular events, as they likely restrict plaque progression. We have recently initiated a preclinical trial implicating oral, selective, small molecule inhibitors of PADI2 (AFM-30a) and PADI4 (JBI-589, GSK199, and GSK484) to suppress chronic low-grade inflammation, aortic atherosclerosis, and aortic valve calcification in ApoE-/- mice. These pharmaceuticals fit the demands of lifelong therapy in terms of route of administration (as they have high bioavailability when used *per os*, in contrast to monoclonal antibodies), expected effects (inhibition of non-specific inflammation and reduced formation of NETs which might provoke thrombosis), and cost (although they are more expensive than antiplatelet drugs, beta-blockers, angiotensin-converting enzyme inhibitors, and statins, they are considerably cheaper than monoclonal antibodies). Nevertheless, their efficacy and safety for impeding the development of atherosclerosis and preventing the occurrence of major adverse cardiovascular events are yet to be confirmed. We suggested hyperlipidemic mice as a relevant model for such a preclinical trial as they rapidly accumulate low-density lipoprotein cholesterol and calcium in the aorta and aortic valve, especially a high-fat diet. However, the trial is ongoing and no conclusive results have been obtained hitherto.

## Figures and Tables

**Table 1:** Clinicopathological features of the patients enrolled on the study.

**Pairwise collected carotid atherosclerotic plaques and adjacent intact arterial segments (n = 14)**	
Male gender (n, %)	9/14 (64.3%)
Age (median ± interquartile range)	66.0 (59.50 – 70.25)
Ischemic stroke (n, %)	4/14 (28.6%)
Arterial hypertension (n, %)	14/14 (100.0%)
Past medical history of myocardial infarction (n, %)	5/14 (35.7%)
Chronic heart failure (n, %)	13/14 (92.8%)
Chronic coronary syndrome (n, %)	12/14 (85.7%)
Past medical history of stroke (n, %)	4/14 (28.6%)
Chronic obstructive pulmonary disease or asthma (n, %)	5/14 (35.7%)
Smoking (n, %)	7/14 (50.0%)
Chronic kidney disease (n, %)	1/14 (7.1%)
Type 2 diabetes mellitus (n, %)	5/14 (35.7%)
Overweight or obesity (n, %)	10/14 (71.4%)
Body mass index (median ± interquartile range)	27.61 (24.28 – 31.13)
Estimated glomerular filtration rate (CKD-EPI 2021, median ± interquartile range)	69.50 (59.50 – 95.00)
Left ventricular ejection fraction (median ± interquartile range)	65.50 (63.00 – 69.00)
Percent stenosis (median ± interquartile range)	77.50 (70.00 – 84.00)
Antiplatelet drugs	11/14 (71.4%)
Beta-blockers	13/14 (92.8%)
Angiotensin-converting enzyme inhibitors	9/14 (64.3%)
Statins	9/14 (64.3%)
Nitrates	1/14 (7.1%)
Angiotensin receptor II blockers	6/14 (42.8%)
Aldosterone antagonists	2/14 (14.2%)
Calcium channel blockers	3/14 (21.4%)
Diuretics	1/14 (7.1%)
Anticoagulants	2/14 (14.2%)

**Table 2:** Sequences of customized primers for RT-qPCR.

**Gene**	**Forward primer sequence**	**Reverse primer sequence**
*CD9*	5′-CCTCACCATGCCGGTCAAAG-3′	5′-GTCCAATGGCAAGGACAGCAA-3′
*CD63*	5′-TTGCTCTACGTCCTCCTGCTG-3′	5′-CACCCACTGCGATGATGACC-3′
*CD81*	5′-ATTTCGTCTTCTGGCTGGCT-3′	5′-CATCATGACAGCGCCCACAG-3′
*PADI2*	5′-GGGCTCTTCCTCACAGCCAT-3′	5′-TCGGTCACAGTTCACCAGCA-3′
*PADI4*	5′-TGCCATCCTGCTGGTGAACT-3′	5′-GGTCTTCGTGCTCAGGGTCA-3′
*ACTB*	5′-CATCGAGCACGGCATCGTCA-3′	5′-TAGCACAGCCTGGACAGCAAC-3′
*GAPDH*	5′-AGCCACATCGCTCAGACAC-3′	5′-GCCCAATACGACCAAATCC-3′
*B2M*	5′-TCCATCCGACATTGAAGTTG-3′	5′-CGGCAGGCATACTCATCTT-3′

**Table 3:** Reverse transcription-quantitative polymerase chain reaction (RT-qPCR) profiling of atherosclerotic plaques and adjacent arterial segments. Shown are genes encoding the proteins which are enriched in extracellular vesicles (*CD9*, *CD63*, and *CD81*) and peptidylarginine deiminases (*PADI2* and *PADI4*). Gene expression has been measured as ΔCt values.

**Sample**	***CD9*, ΔCt**	***CD63*, ΔCt**	***CD81*, ΔCt**	***PADI2*, ΔCt**	***PADI4*, ΔCt**
Atherosclerotic plaques					
1	0,061	0,184	0,118	-	0,0002
2	0,028	0,034	0,015	-	0,0002
3	0,045	0,046	0,016	-	0,0001
4	-	0,116	0,035	-	-
5	0,263	0,115	0,094	-	-
6	0,055	0,132	0,131	-	0,0004
7	0,040	0,085	0,127	-	0,0000
8	0,061	0,113	0,077	-	0,0015
Mean ΔCt	0,079	0,103	0,077	-	0,0004
Adjacent arterial segments					
1	0,035	0,036	0,042	-	0,0002
2	0,054	0,039	0,013	-	0,0002
3	0,032	0,021	0,014	-	0,0003
4	0,100	0,081	0,060	-	0,0028
5	-	0,088	0,055	-	-
6	0,122	0,414	0,271	-	0,0007
7	0,027	0,083	0,069	-	0,0005
8	0,081	-	0,024	-	-
Mean ΔCt	0,065	0,109	0,069	-	0,0008
Liver					
ΔCt	0,001	0,645	0,112	0,001	0,658

## Data Availability

The mass spectrometry proteomics data have been deposited to the ProteomeXchange Consortium *via* the PRIDE partner repository with the dataset identifier PXD035173. Other data presented in this study are available on request from the corresponding author.

## LIST OF ABBREVIATIONS

PADI: Peptidylarginine Deiminase
CVD: Cardiovascular Disease
NETs: Neutrophil Extracellular Traps
MPO: Myeloperoxidase
ACPAs: Anti-citrullinated protein Anti-bodies
