# Supplementary material for: Citrullination Accompanies the Development of Carotid Atherosclerotic Plaques
Source: Curr Med Chem. 2024 Oct 17;33(9):1730–58. doi: 10.2174/0109298673327175240929222308 (PMC13223441; doi:10.2174/0109298673327175240929222308)
Supplement: Supplementary file 1 [file CMC-33-9-1730_SD1.pdf]

Supplementary Material

Citrullination Accompanies the Development of Carotid Atherosclerotic Plaques

Anastasia Kanonykina[1], Elena Velikanova[1], Victoria Markova[1], Leo Bogdanov[1], Daria Shishkova[1], Amin Shabaev[1], Maxim Sinitsky[1], Anna Sinitskaya[1], Alyona Poddubnyak[1], Anastasia Lazebnaya[1], Alexander Stepanov[1], Arina Tyurina[1], Arseniy Lobov[2], Bozhana Zainullina[3], Arseniy Yuzhalin[1] and Anton Kutikhin[1,*]

[1]Department of Experimental Medicine, Research Institute for Complex Issues of Cardiovascular Diseases, 6 Sosnovy Boulevard, 650002, Kemerovo, Russia; [2]Laboratory of Regenerative Biomedicine, Institute of Cytology of the RAS, 4 Tikhoretskiy Prospekt, St. Petersburg, 194064, Russia; [3]Centre for Molecular and Cell Technologies, St. Petersburg State University, Universitetskaya Embankment, 7/9, 199034, St. Petersburg, Russia

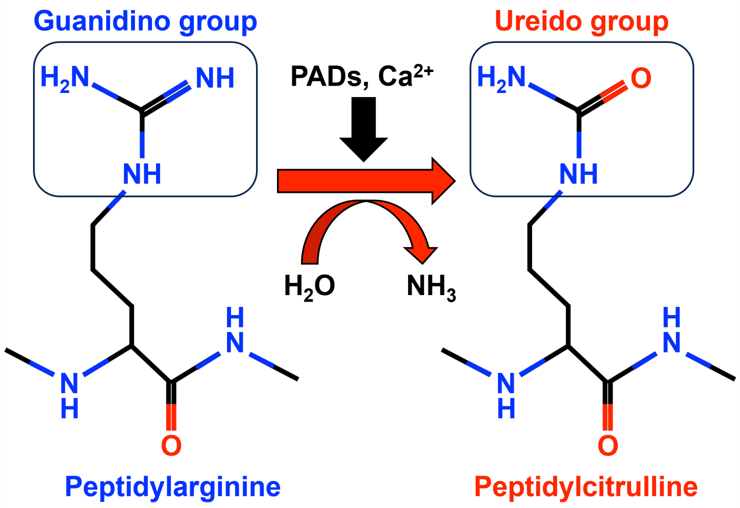

Fig. (S1). The structures of arginine and citrulline and the mechanism of arginine-to-citrulline conversion.

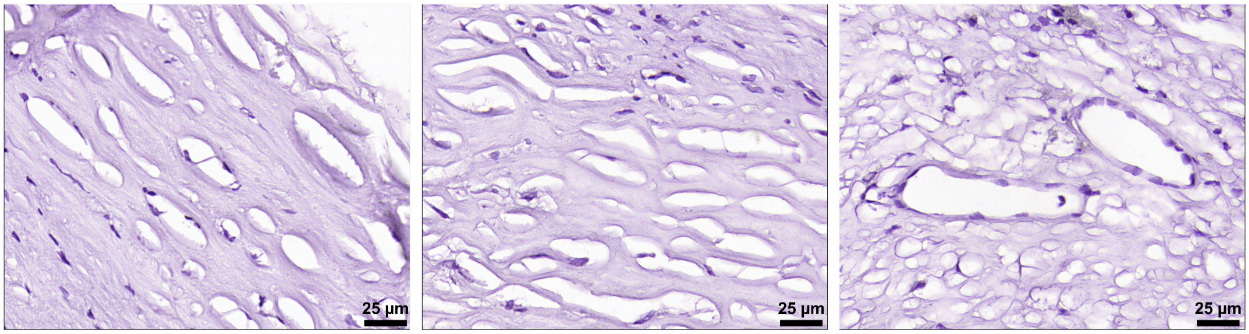

Fig. (S2). Staining of atherosclerotic plaques with haematoxylin and eosin. Microvessels variable in size and shape. Magnification: ×800, scale bar: 25 µm.

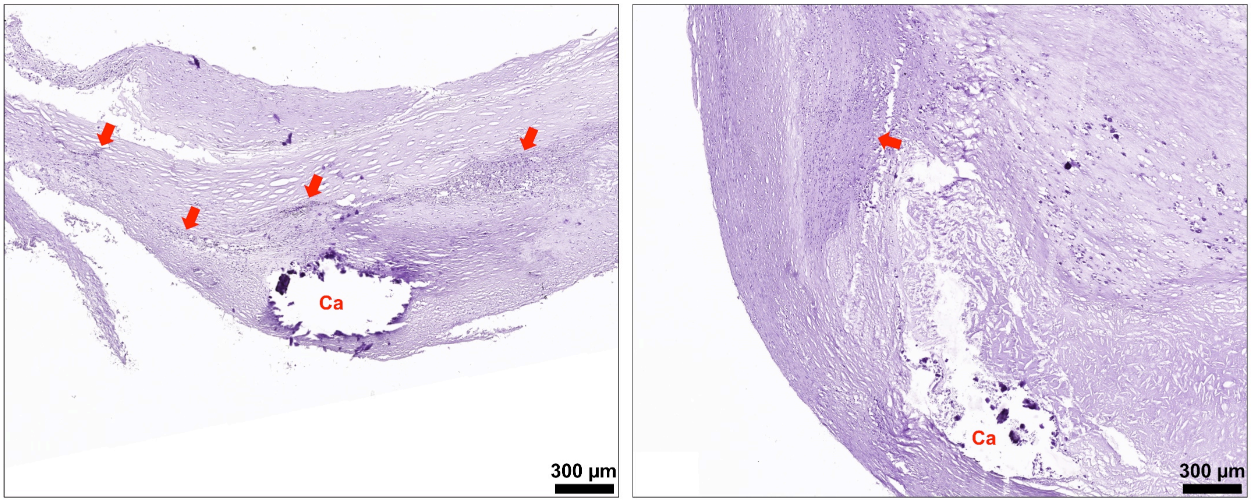

Fig. (S3). Staining of atherosclerotic plaques with haematoxylin and eosin. Calcifications and adjacent leukocyte infiltrations. Magnification: ×60, scale bar: 300 μm.

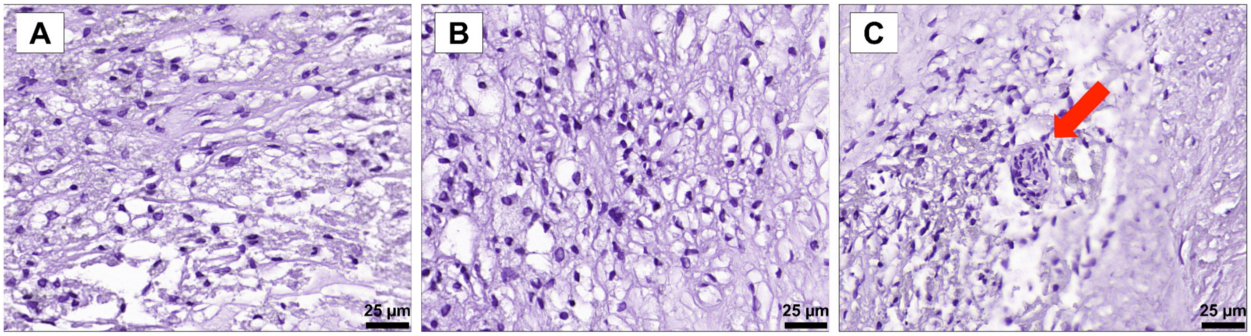

Fig. (S4). Staining of atherosclerotic plaques with haematoxylin and eosin. Leukocyte infiltrations. A. Moderate leukocyte density near the calcifications. B. High leukocyte density at the border between neointima and tunica media. C. High leukocyte density and leukocyte clusters at the border between neointima and tunica media. Magnification: ×800, scale bar: 25 μm.

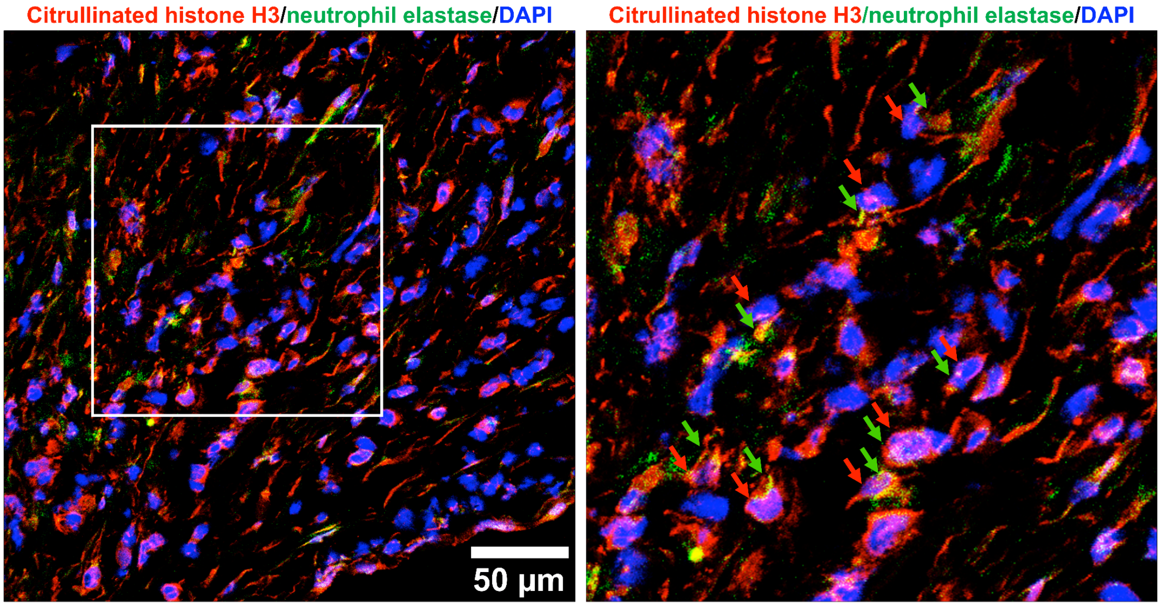

Fig. (S5). Immunofluorescence staining of neointima and adjacent tunica media for citrullinated histone H3 (red colour) and neutrophil elastase (green colour). Nuclei are counterstained with DAPI (blue colour). Magnification: ×400, scale bar: 50 μm.

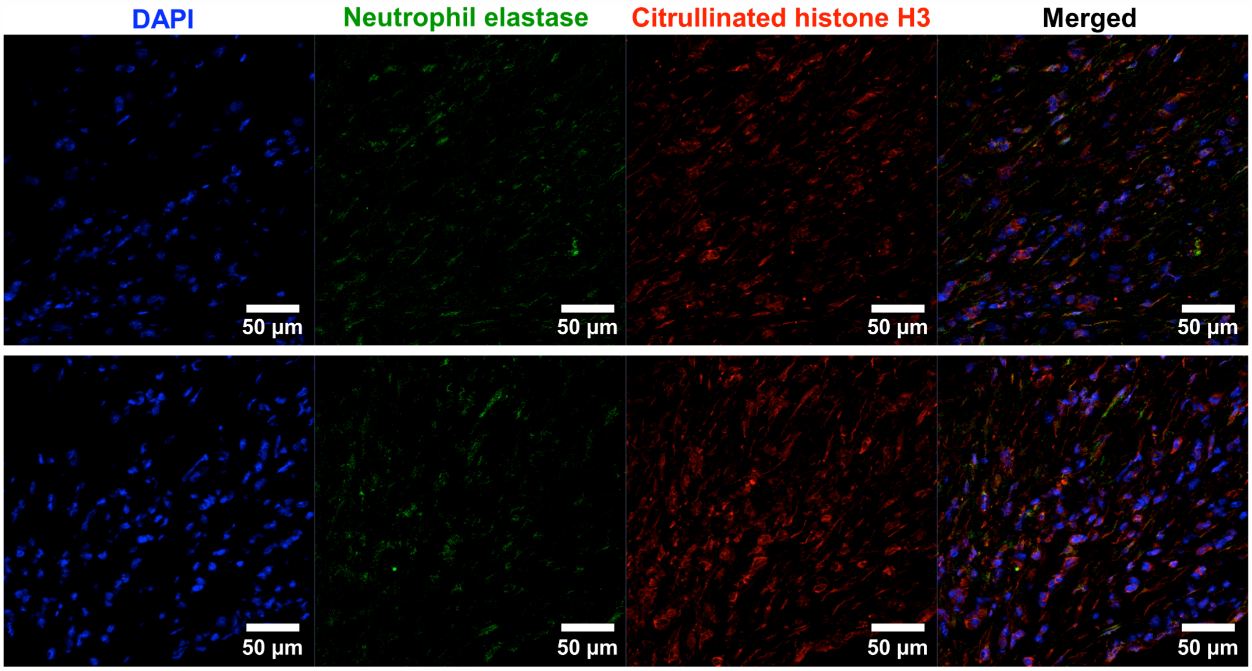

Fig. (S6). Channel split for DAPI (blue colour), neutrophil elastase (green colour), and citrullinated histone H3 (red colour), immunofluorescence staining (merged image is provided at the right side). Magnification: ×400, scale bar: 50 μm.

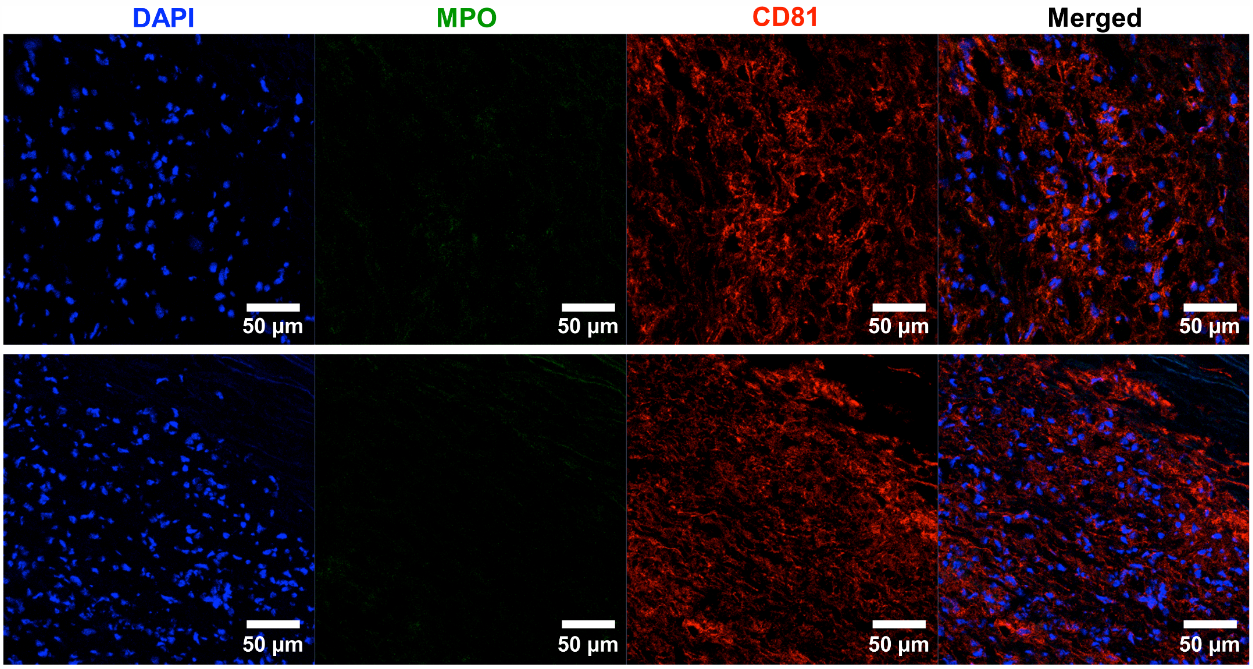

Fig. (S7). Channel split for DAPI (blue colour), MPO (green colour), and CD81 (red colour), immunofluorescence staining (merged image is provided at the right side). Magnification: ×400, scale.

Table S1. Biological processes involving the proteins which are upregulated in either carotid atherosclerotic plaques or adjacent arterial segments (annotated according to the Gene Ontology Biological Process database). DEPs: differentially expressed proteins.

GO term (Biological Process)	Carotid plaques (fold change, observed versus expected numbers of DEPs)	Adjacent arterial segments (fold change, observed versus expected numbers of DEPs)
**Immune response**		
Activation of innate immune response	8.24 (8 vs 0.97)<br>P08631 (HCK)<br>Q96PK6 (RBM14)<br>Q9Y6K5 (OAS3)<br>P41218 (MNDA)<br>P43243 (MATR3)<br>P09429 (HMGB1)<br>P16885 (PLCG2)<br>O00203 (AP3B1)	-
Acute inflammatory response	8.04 (8 vs 1.00)<br>P08697 (SERPINF2)<br>P00738 (HP)<br>P02763 (ORM1)<br>P02765 (AHSG)<br>P00966 (ASS1)<br>P09429 (HMGB1)<br>P12931 (SRC)<br>Q99623 (PHB2)	-
Inflammatory response	2.93 (19 vs 6.49)<br>P08631 (HCK)<br>P01031 (C5)<br>P09429 (HMGB1)<br>P32119 (PRDX2)<br>Q9UKK3 (PARP4)<br>P06702 (S100A9)<br>Q8NE71 (ABCF1)<br>O00203 (AP3B1)<br>P05109 (S100A8)<br>P01911 (HLA-DRB1)<br>P08697 (SERPINF2)<br>P00738 (HP)<br>P02763 (ORM1)<br>P01042 (KNG1)<br>P02765 (AHSG)<br>Q9NZC2 (TREM2)<br>P00966 (ASS1)<br>P12931 (SRC)<br>Q99623 (PHB2)	-
Activation of immune response	4.22 (19 vs 4.50)<br>P08631 (HCK)<br>P19174 (PLCG1)<br>Q96PK6 (RBM14)<br>Q9Y6K5 (OAS3)<br>P41218 (MNDA)<br>P01031 (C5)<br>P43243 (MATR3)<br>P09429 (HMGB1)<br>P02748 (C9)<br>P16885 (PLCG2)<br>O00203 (AP3B1)<br>P36980 (CFHR2)<br>P12931 (SRC)<br>P01911 (HLA-DRB1)<br>Q99623 (PHB2)<br>P01859 (IGHG2)<br>P48740 (MASP1)<br>P02765 (AHSG)	6.46 (14 vs 2.17)<br>P46379 (BAG6)<br>Q92496 (CFHR4)<br>P09429 (HMGB1)<br>P20851 (C4BPB)<br>P15529 (CD46)<br>P46109 (CRKL)<br>P08311 (CTSG)<br>P08246 (ELANE)<br>P16671 (CD36)<br>P38571 (LIPA)<br>P07437 (TUBB)<br>P60953 (CDC42)<br>Q96C90 (PPP1R14B)<br>P68371 (TUBB4B)

	Q9NZC2 (TREM2)	
Immune response-activating signal transduction	3.86 (14 vs 3.62)<br>P08631 (HCK)<br>P19174 (PLCG1)<br>Q9Y6K5 (OAS3)<br>P41218 (MNDA)<br>P16885 (PLCG2)<br>O00203 (AP3B1)<br>P12931 (SRC)<br>P01911 (HLA-DRB1)<br>Q99623 (PHB2)<br>P01859 (IGHG2)<br>O00203 (AP3B1)<br>P48740 (MASP1)<br>P02765 (AHSG)<br>Q9NZC2 (TREM2)	6.86 (12 vs 1.75)<br>P46379 (BAG6)<br>Q92496 (CFHR4)<br>P09429 (HMGB1)<br>P20851 (C4BPB)<br>P15529 (CD46)<br>P46109 (CRKL)<br>P08311 (CTSG)<br>P08246 (ELANE)<br>P16671 (CD36)<br>P38571 (LIPA)<br>P07437 (TUBB)<br>P60953 (CDC42)
Positive regulation of immune response	2.73 (20 vs 7.32)<br>P08631 (HCK)<br>P19174 (PLCG1)<br>Q96PK6 (RBM14)<br>Q9Y6K5 (OAS3)<br>P41218 (MNDA)<br>P01031 (C5)<br>P43243 (MATR3)<br>P09429 (HMGB1)<br>P02748 (C9)<br>P16885 (PLCG2)<br>Q8IWB7 (WDFY1)<br>O00203 (AP3B1)<br>P36980 (CFHR2)<br>P12931 (SRC)<br>P01911 (HLA-DRB1)<br>Q99623 (PHB2)<br>P01859 (IGHG2)<br>P48740 (MASP1)<br>P02765 (AHSG)<br>Q9NZC2 (TREM2)	4.53 (16 vs 3.53)<br>P46379 (BAG6)<br>Q92496 (CFHR4)<br>P09429 (HMGB1)<br>P20851 (C4BPB)<br>P15529 (CD46)<br>P46109 (CRKL)<br>P08311 (CTSG)<br>P08246 (ELANE)<br>P16671 (CD36)<br>P38571 (LIPA)<br>P07437 (TUBB)<br>P60953 (CDC42)<br>Q96C90 (PPP1R14B)<br>P68371 (TUBB4B)
Innate immune response	2.56 (26 vs 10.16)<br>P08631 (HCK)<br>Q96PK6 (RBM14)<br>P46934 (NEDD4)<br>Q9BWS9 (CHID1)<br>Q9Y6K5 (OAS3)<br>Q8IYM9 (TRIM22)<br>P01031 (C5)<br>P43243 (MATR3)<br>P09429 (HMGB1)<br>P02748 (C9)<br>P29590 (PML)<br>P16885 (PLCG2)<br>P06702 (S100A9)<br>P12931 (SRC)<br>P05109 (S100A8)<br>Q99623 (PHB2)<br>P19525 (EIF2AK2)<br>P48740 (MASP1)<br>P40429 (RPL13A)<br>P00966 (ASS1)<br>P01911 (HLA-DRB1)<br>P01859 (IGHG2)<br>P01042 (KNG1)<br>P41218 (MNDA)<br>P02765 (AHSG)<br>Q9NZC2 (TREM2)	3.27 (16 vs 4.90)<br>P46379 (BAG6)<br>Q92496 (CFHR4)<br>P09429 (HMGB1)<br>P20851 (C4BPB)<br>P15529 (CD46)<br>P46109 (CRKL)<br>P08311 (CTSG)<br>P08246 (ELANE)<br>P16671 (CD36)<br>P38571 (LIPA)<br>P07437 (TUBB)<br>P60953 (CDC42)<br>Q96C90 (PPP1R14B)<br>P68371 (TUBB4B)
Humoral immune response	3.86 (16 vs 4.14)	7.01 (14 vs 2.00)

	P01031 (C5)<br>P01619 (IKGV3-20)<br>P02748 (C9)<br>P06702 (S100A9)<br>P24158 (PRTN3)<br>P36980 (CFHR2)<br>P01911 (HLA-DRB1)<br>P01859 (IGHG2)<br>P48740 (MASP1)<br>Q9NZC2 (TREM2)<br>Q96PK6 (RBM14)<br>Q9Y6K5 (OAS3)<br>P43243 (MATR3)<br>P09429 (HMGB1)<br>P05109 (S100A8)<br>Q99623 (PHB2)	P46379 (BAG6)<br>Q92496 (CFHR4)<br>P09429 (HMGB1)<br>P20851 (C4BPB)<br>P15529 (CD46)<br>P46109 (CRKL)<br>P08311 (CTSG)<br>P08246 (ELANE)<br>P16671 (CD36)<br>P38571 (LIPA)<br>P07437 (TUBB)<br>P60953 (CDC42)<br>Q96C90 (PPP1R14B)<br>P02775 (PPBP)
Positive regulation of B cell activation	-	9.93 (10 vs 1.01)<br>P46379 (BAG6)<br>Q92496 (CFHR4)<br>P09429 (HMGB1)<br>P20851 (C4BPB)<br>P08311 (CTSG)<br>P08246 (ELANE)<br>P16671 (CD36)<br>P60953 (CDC42)<br>Q96C90 (PPP1R14B)<br>P02775 (PPBP)
Immunoglobulin production	-	7.38 (7 vs 0.95)<br>P04433 (IGKV3-11)<br>P01614 (IGKV2D-40)<br>A0A087WSZ0 (IGKV1D-8)<br>P01611 (IGKV1D-12)<br>P01602 (IGKV1-5)<br>A0A0B4J1U3 (IGLV1-36)<br>A0A0A0MT36 (IGKV6D-21)
Lymphocyte mediated immunity	-	8.85 (13 vs 1.47)<br>P07437 (TUBB)<br>A0A0C4DH36 (IGHV3-38)<br>A0A0B4J1U7 (IGHV6-1)<br>P01817 (IGHV2-5)<br>A0A0J9YX35 (IGHV3-64D)<br>P20851 (C4BPB)<br>A0A0C4DH34 (IGHV4-28)<br>A0A0B4J1X5 (IGHV3-74)<br>P68371 (TUBB4B)<br>A0A0C4DH35 (IGHV3-35)<br>A0A0B4J1V2 (IGHV2-26)<br>A0A0A0MS15 (IGHV3-49)<br>P01764 (IGHV3-23)
Positive regulation of lymphocyte activation	-	5.35 (13 vs 2.43)<br>P09429 (HMGB1)<br>P15529 (CD46)<br>P61586 (RHOA)<br>A0A0C4DH36 (IGHV3-38)<br>A0A0B4J1U7 (IGHV6-1)<br>P01817 (IGHV2-5)<br>A0A0J9YX35 (IGHV3-64D)<br>A0A0C4DH34 (IGHV4-28)<br>A0A0B4J1X5 (IGHV3-74)<br>A0A0C4DH35 (IGHV3-35)<br>A0A0B4J1V2 (IGHV2-26)<br>A0A0A0MS15 (IGHV3-49)<br>P01764 (IGHV3-23)
Adaptive immune response	-	4.92 (19 vs 3.86)<br>P04433 (IGKV3-11)

		P01614 (IGKV2D-40)<br>A0A0C4DH36 (IGHV3-38)<br>A0A0B4J1U7 (IGHV6-1)<br>P01817 (IGHV2-5)<br>A0A0J9YX35 (IGHV3-64D)<br>P09429 (HMGB1)<br>A0A087WSZ0 (IGKV1D-8)<br>P20851 (C4BPB)<br>P01611 (IGKV1D-12)<br>P15529 (CD46)<br>A0A0C4DH34 (IGHV4-28)<br>P01602 (IGKV1-5)<br>A0A0B4J1X5 (IGHV3-74)<br>A0A0B4J1U3 (IGLV1-36)<br>A0A0C4DH35 (IGHV3-35)<br>A0A0A0MT36 (IGKV6D-21)<br>A0A0B4J1V2 (IGHV2-26)<br>A0A0A0MS15 (IGHV3-49)<br>P01764 (IGHV3-23)
**Response to bacteria**		
Complement activation	5.85 (10 vs 1.71)<br>P01031 (C5)<br>P02748 (C9)<br>P36980 (CFHR2)<br>P01859 (IGHG2)<br>P48740 (MASP1)<br>P04430 (IGKV1-16)<br>P22792 (CPN2)<br>P80748 (IGLV3-21)<br>P01766 (IGHV3-13)<br>P06310 (IGKV2-30)	13.36 (11 vs 0.82)<br>Q92496 (CFHR4)<br>P20851 (C4BPB)<br>P15529 (CD46)
Response to lipopolysaccharide	3.95 (15 vs 3.80)<br>P08631 (HCK)<br>P05164 (MPO)<br>Q9NRQ2 (PLSCR4)<br>P30536 (TSPO)<br>Q92769 (HDAC2)<br>P09429 (HMGB1)<br>P32119 (PRDX2)<br>P16885 (PLCG2)<br>P06702 (S100A9)<br>P12931 (SRC)<br>P05109 (S100A8)<br>P14780 (MMP9)<br>P40429 (RPL13A)<br>Q9NZC2 (TREM2)<br>P00966 (ASS1)	-
Defense response to bacterium	3.12 (14 vs 4.49)<br>Q9Y6K5 (OAS3)<br>P05164 (MPO)<br>P01619 (IKGV3-20)<br>P06702 (S100A9)<br>P05109 (S100A8)<br>P00738 (HP)<br>P01859 (IGHG2)<br>Q9NZC2 (TREM2)<br>P01031 (C5)<br>P02748 (C9)<br>P36980 (CFHR2)<br>P48740 (MASP1)<br>P32119 (PRDX2)<br>P40429 (RPL13A)	6.47 (14 vs 2.16)<br>P46379 (BAG6)<br>Q92496 (CFHR4)<br>P09429 (HMGB1)<br>P20851 (C4BPB)<br>P15529 (CD46)<br>P46109 (CRKL)<br>P08311 (CTSG)<br>P08246 (ELANE)<br>P16671 (CD36)<br>P38571 (LIPA)<br>P07437 (TUBB)<br>P60953 (CDC42)<br>Q96C90 (PPP1R14B)<br>P02775 (PPBP)
**Oxidative stress**		
Respiratory burst involved in defense response	40.69 (4 vs 0.1)<br>P08631 (HCK)	-

	P05164 (MPO)<br>P32119 (PRDX2)<br>Q9NZC (TREM2)	
Respiratory burst	13.02 (4 vs 0.31)<br>P08631 (HCK)<br>P05164 (MPO)<br>P32119 (PRDX2)<br>Q9NZC (TREM2)	-
Response to oxygen-containing compound	2.08 (39 vs 18.73)<br>P08631 (HCK)<br>P35222 (CTNNB1)<br>P05164 (MPO)<br>P05090 (APOD)<br>P01019 (AGT)<br>P09471 (GNAO1)<br>P42229 (STAT5A)<br>Q6PKG0 (LARP1)<br>Q9NRQ2 (PLSCR4)<br>P30536 (TSPO)<br>Q92769 (HDAC2)<br>P09429 (HMGB1)<br>Q9P2J5 (LARS1)<br>P32119 (PRDX2)<br>Q86X55 (CARM1)<br>P08709 (F7)<br>P16885 (PLCG2)<br>P17302 (GJA1)<br>P21589 (NT5E)<br>P06702 (S100A9)<br>P12931 (SRC)<br>P05109 (S100A8)<br>Q92572 (AP3S1)<br>Q96SB3 (PPP1R9B)<br>Q8N573 (OXR1)<br>P46060 (RANGAP1)<br>Q99623 (PHB2)<br>P00738 (HP)<br>P14780 (MMP9)<br>Q969T9 (WBP2)<br>P40429 (RPL13A)<br>Q86UE4 (MTDH)<br>Q6P9B6 (MEAK7)<br>P15104 (GLUL)<br>Q9NZC2 (TREM2)<br>P00966 (ASS1)<br>Q8TAL6 (FIBIN)<br>P41218 (MNDA)<br>Q96PK6 (RBM14)	-
**Phagocytosis**		
Lysosomal transport	6.62 (10 vs 1.51)<br>P46934 (NEDD4)<br>Q96FZ7 (CHMP6)<br>Q9NZN3 (EHD3)<br>Q5VIR6 (VPS53)<br>Q9UN37 (VPN4A)<br>O00203 (AP3B1)<br>Q9Y2T2 (AP3M1)<br>Q9P2R3 (ANKFY1)<br>Q6IQ22 (RAB12)<br>Q86VS8 (HOOK3)	-
Regulation of phagocytosis	6.38 (8 vs 1.25)<br>P08631 (HCK)<br>P05164 (MPO)<br>P09429 (HMGB1)<br>P16885 (PLCG2)	-

	P24158 (PRTN3)<br>Q92614 (MYO18A)<br>P02765 (AHSG)<br>Q9NZC2 (TREM2)	
Phagocytosis, engulfment	6.21 (10 vs 1.61)<br>P08631 (HCK)<br>P05164 (MPO)<br>P09429 (HMGB1)<br>P16885 (PLCG2)<br>P24158 (PRTN3)<br>Q92614 (MYO18A)<br>P02765 (AHSG)<br>Q9NZC2 (TREM2)<br>Q9UBP9 (GULP1)<br>Q92556 (ELMO1)	15.46 (12 vs 0.78)<br>P46379 (BAG6)<br>Q92496 (CFHR4)<br>P09429 (HMGB1)<br>P20851 (C4BPB)<br>P15529 (CD46)<br>P46109 (CRKL)<br>P08311 (CTSG)<br>P08246 (ELANE)<br>P16671 (CD36)<br>P38571 (LIPA)<br>P07437 (TUBB)<br>P60953 (CDC42)
Phagocytosis, recognition	6.14 (8 vs 1.30)<br>P08631 (HCK)<br>P05164 (MPO)<br>P09429 (HMGB1)<br>P16885 (PLCG2)<br>P24158 (PRTN3)<br>Q92614 (MYO18A)<br>P02765 (AHSG)<br>Q9NZC2 (TREM2)	17.51 (11 vs 0.63)<br>P46379 (BAG6)<br>Q92496 (CFHR4)<br>P09429 (HMGB1)<br>P20851 (C4BPB)<br>P15529 (CD46)<br>P46109 (CRKL)<br>P08311 (CTSG)<br>P08246 (ELANE)<br>P16671 (CD36)<br>P38571 (LIPA)<br>P07437 (TUBB)
Phagocytosis	4.86 (15 vs 3.08)<br>P08631 (HCK)<br>P05164 (MPO)<br>P09429 (HMGB1)<br>P16885 (PLCG2)<br>P24158 (PRTN3)<br>Q92614 (MYO18A)<br>P02765 (AHSG)<br>Q9NZC2 (TREM2)<br>Q9UBP9 (GULP1)<br>Q92556 (ELMO1)<br>Q96JJ3 (ELMO2)<br>P12931 (SRC)<br>Q9UNF0 (PACSIN2)<br>P46934 (NEDD4)<br>Q9NZN3 (EHD3)	10.09 (15 vs 1.49)<br>P46379 (BAG6)<br>P50570 (DNM2)<br>Q92496 (CFHR4)<br>P09429 (HMGB1)<br>P20851 (C4BPB)<br>P15529 (CD46)<br>P46109 (CRKL)<br>P08311 (CTSG)<br>P08246 (ELANE)<br>P16671 (CD36)<br>P38571 (LIPA)<br>P07437 (TUBB)<br>P60953 (CDC42)<br>Q96C90 (PPP1R14B)<br>P02775 (PPBP)
Endocytosis	3.29 (21 vs 6.38)<br>P08631 (HCK)<br>Q9UNF0 (PACSIN2)<br>P46934 (NEDD4)<br>Q9UBP9 (GULP1)<br>Q96JJ3 (ELMO2)<br>Q9NZN3 (EHD3)<br>Q9NZQ3 (NCKIPSD)<br>Q92556 (ELMO1)<br>Q9Y6W5 (WASF2)<br>P09429 (HMGB1)<br>Q99961 (SH3GL1)<br>P12931 (SRC)<br>O00203 (AP3B1)<br>Q9Y2T2 (AP3M1)<br>Q92572 (AP3S1)<br>Q9P2R3 (ANKFY1)<br>O00291 (HIP1)<br>P02765 (AHSG)<br>Q9NZC2 (TREM2)	4.88 (15 vs 3.08)<br>P08246 (ELANE)<br>P16671 (CD36)<br>Q96CW1 (AP2M1)<br>P60953 (CDC42)<br>P09429 (HMGB1)<br>P50570 (DNM2)<br>P38571 (LIPA)<br>P15311 (EZR)<br>P46109 (CRKL)<br>P08311 (CTSG)<br>P07437 (TUBB)<br>P46379 (BAG6)<br>Q92496 (CFHR4)<br>P20851 (C4BPB)<br>P15529 (CD46)

	P16885 (PLCG2)<br>P24158 (PRTN3)	
**Biosynthetic processes**		
Translation	3.67 (17 vs 4.63)<br>Q99613 (EIF3C)<br>E9PAV3 (NACA)<br>Q6PKG0 (LARP1)<br>Q9P2J5 (LARS1)<br>Q9Y295 (DRG1)<br>Q9UKV8 (AGO2)<br>Q8NE71 (ABCF1)<br>P27635 (RPL10)<br>P42766 (RPL35)<br>Q86SH2 (ZAR1)<br>Q9BRX2 (PELO)<br>P26373 (RPL13)<br>P26639 (TARS1)<br>P15880 (RPS2)<br>P46781 (RPS9)<br>Q96JB5 (CDK5RAP3)<br>P19525 (EIF2AK2)	-
Peptide biosynthetic process	3.42 (17 vs 4.96)<br>Q99613 (EIF3C)<br>E9PAV3 (NACA)<br>Q6PKG0 (LARP1)<br>Q9P2J5 (LARS1)<br>Q9Y295 (DRG1)<br>Q9UKV8 (AGO2)<br>Q8NE71 (ABCF1)<br>P27635 (RPL10)<br>P42766 (RPL35)<br>Q86SH2 (ZAR1)<br>Q9BRX2 (PELO)<br>P26373 (RPL13)<br>P26639 (TARS1)<br>P15880 (RPS2)<br>P46781 (RPS9)<br>Q96JB5 (CDK5RAP3)<br>P19525 (EIF2AK2)	-
Amide biosynthetic process	2.82 (18 vs 6.39)<br>Q99613 (EIF3C)<br>E9PAV3 (NACA)<br>Q6PKG0 (LARP1)<br>Q9P2J5 (LARS1)<br>Q9Y295 (DRG1)<br>Q9UKV8 (AGO2)<br>Q8NE71 (ABCF1)<br>P27635 (RPL10)<br>P42766 (RPL35)<br>Q86SH2 (ZAR1)<br>Q9BRX2 (PELO)<br>P26373 (RPL13)<br>P26639 (TARS1)<br>P15880 (RPS2)<br>P46781 (RPS9)<br>Q96JB5 (CDK5RAP3)<br>P19525 (EIF2AK2)<br>P40429 (RPL13A)	-
Cellular macromolecule biosynthetic process	2.45 (23 vs 9.39)<br>Q99613 (EIF3C)<br>E9PAV3 (NACA)<br>Q6PKG0 (LARP1)<br>Q9P2J5 (LARS1)<br>Q9Y295 (DRG1)<br>Q9UKV8 (AGO2)	-

	Q8NE71 (ABCF1)<br>P27635 (RPL10)<br>P42766 (RPL35)<br>Q86SH2 (ZAR1)<br>Q9BRX2 (PELO)<br>P26373 (RPL13)<br>P26639 (TARS1)<br>P15880 (RPS2)<br>P46781 (RPS9)<br>Q96JB5 (CDK5RAP3)<br>P19525 (EIF2AK2)<br>P40429 (RPL13A)	
Regulation of cellular component bio-genesis	2.38 (28 vs 11.76)<br>P08631 (HCK)<br>Q96PK6 (RBM14)<br>P35222 (CTNNB1)<br>Q96FZ7 (CHMP6)<br>P35611 (ADD1)<br>P05090 (APOD)<br>P01019 (AGT)<br>Q7Z3B1 (NEGR1)<br>Q8TCU4 (ALMS1)<br>Q9UN37 (VPS4A)<br>Q9BPX5 (ARPC5L)<br>Q9Y6W5 (WASF2)<br>Q9BTW9 (TBCD)<br>P09429 (HMGB1)<br>Q9Y295 (DRG1)<br>P16885 (PLCG2)<br>Q92783 (STAM)<br>Q8TBX8 (PIP4K2C)<br>P50281 (MMP14)<br>P12931 (SRC)<br>Q8N392 (ARHGAP18)<br>Q9UNN5 (FAF1)<br>P08697 (SERPINF2)<br>Q9UEY8 (ADD3)<br>Q8NCA5 (FAM98A)<br>P19525 (EIF2AK2)<br>P40429 (RPL13A)<br>Q9NZC2 (TREM2)	-
**Extracellular vesicles**		
Clathrin-coated vesicle cargo loading	34.88 (3 vs 0.09)<br>O00203 (AP3B1)<br>Q92572 (AP3S1)<br>Q9Y2T2 (AP3M1)	-
Vesicle budding from membrane	6.42 (6 vs 0.93)<br>Q96FZ7 (CHMP6)<br>Q9UN37 (VPS4A)<br>Q9Y296 (TRAPPC4)<br>Q92614 (MYO18A)<br>Q92572 (AP3S1)<br>Q9Y2T2 (AP3M1)	-
Vesicle organisation	3.34 (13 vs 3.90)<br>Q96FZ7 (CHMP6)<br>Q9UN37 (VPS4A)<br>Q92783 (STAM)<br>Q9Y296 (TRAPPC4)<br>O00203 (AP3B1)<br>Q92614 (MYO18A)<br>Q92572 (AP3S1)<br>Q9Y2T2 (AP3M1)<br>Q9P2R3 (ANKFY1)<br>P07093 (SERPINE2)<br>Q86VS8 (HOOK3)	-

	Q8IYI6 (EXOC8)<br>P48426 (PIP4K2A)	
Vesicle-mediated transport	2.60 (43 vs 16.54)<br>P08631 (HCK)<br>Q9UNF0 (PACSIN2)<br>P46934 (NEDD4)<br>P35222 (CTNNB1)<br>Q96FZ7 (CHMP6)<br>Q9UBP9 (GULP1)<br>Q96JJ3 (ELMO2)<br>Q9NZN3 (EHD3)<br>Q9UJD0 (RIMS3)<br>Q5VIR6 (VPS53)<br>P09471 (GNAO1)<br>P19022 (CDH2)<br>Q9NZQ3 (NCKIPSD)<br>Q8TCU4 (ALMS1)<br>Q9UN37 (VPS4A)<br>Q92556 (ELMO1)<br>Q9Y6W5 (WASF2)<br>P09429 (HMGB1)<br>Q92783 (STAM)<br>Q9Y296 (TRAPPC4)<br>O00203 (AP3B1)<br>Q99961 (SH3GL1)<br>Q92614 (MYO18A)<br>P12931 (SRC)<br>Q92572 (AP3S1)<br>Q9Y2T2 (AP3M1)<br>Q9P2R3 (ANKFY1)<br>P40616 (ARL1)<br>Q6IQ22 (RAB12)<br>Q9UPT5 (EXOC7)<br>O00291 (HIP1)<br>Q86VS8 (HOOK3)<br>Q8TDW5 (SYTL5)<br>Q8IYI6 (EXOC8)<br>P02765 (AHSG)<br>Q9NZC2 (TREM2)<br>P48426 (PIP4K2A)	2.88 (23 vs 7.98)<br>Q7Z3J2 (VPS35L)<br>P08246 (ELANE)<br>Q9UBF2 (COPG2)<br>P16671 (CD36)<br>Q96CW1 (AP2M1)<br>P07355 (ANXA2)<br>P60953 (CDC42)<br>P09429 (HMGB1)<br>Q9UJY5 (GGA1)<br>P50570 (DNM2)<br>Q8WUX9 (CHMP7)<br>P38571 (LIPA)<br>P15311 (EZR)
**Miscellaneous**		
Extracellular matrix disassembly	-	17.95 (5 vs 0.28)<br>P08311 (CTSG)<br>Q96AQ6 (PBXIP1)<br>P03956 (MMP1)<br>P08246 (ELANE)<br>Q99542 (MMP19)
Regulation of coagulation	6.60 (6 vs 0.91)<br>P00747 (PLG)<br>P32119 (PRDX2)<br>P08709 (F7)<br>P08697 (SERPINF2)<br>P07093 (SERPINE2)<br>P01042 (KNG1)	-
Response to lipid	2.40 (24 vs 9.99)<br>P08631 (HCK)<br>P46934 (NEDD4)<br>P35222 (CTNNB1)<br>P05164 (MPO)<br>Q9NRQ2 (PLSCR4)<br>P30536 (TSPO)<br>Q92769 (HDAC2)<br>P09429 (HMGB1)<br>P32119 (PRDX2)<br>P08709 (F7)	-

	P16885 (PLCG2)<br>P06702 (S100A9)<br>P12931 (SRC)<br>P05109 (S100A8)<br>Q96SB3 (PPP1R9B)<br>Q99623 (PHB2)<br>P14780 (MMP9)<br>Q969T9 (WBP2)<br>P40429 (RPL13A)<br>Q86UE4 (MTDH)<br>Q9NZC2 (TREM2)<br>P00966 (ASS1)<br>Q8TAL6 (FIBIN)<br>P02765 (AHSG)	
Positive regulation of cell death	2.81 (20 vs 7.11)	-

Table S2. Biological processes involving the proteins which are upregulated in either carotid atherosclerotic plaques or adjacent arterial segments (annotated according to the Reactome database). DEPs: differentially expressed proteins.

Reactome term	Carotid plaques<br>(fold change, observed versus expected numbers of DEPs)	Adjacent arterial segments<br>(fold change, observed versus expected numbers of DEPs)
**Immune response**		
Role of phospholipids in phagocytosis	10.17 (11 vs 1.08)<br>P19174 (PLCG1)<br>P04430 (IGKV1-16)<br>P80748 (IGLV3-21)<br>P01766 (IGHV3-13)<br>P01619 (IGKV3-20)<br>P06310 (IGKV2-30)<br>P06331 (IGHV4-34)<br>P16885 (PLCG2)<br>P01859 (IGHG2)<br>P01780 (IGHV3-7)<br>P01764 (IGHV3-23)	11.51 (6 vs 0.52)<br>P04433 (IGKV3-11)<br>P01614 (IGKV2D-40)<br>P01817 (IGHV2-5)<br>P01611 (IGKV1D-12)<br>P01602 (IGKV1-5)<br>P01764 (IGHV3-23)
Regulation of complement cascade	9.62 (13 vs 1.35)<br>P04430 (IGKV1-16)<br>P22792 (CPN2)<br>P80748 (IGLV3-21)<br>P01766 (IGHV3-13)<br>P01031 (C5)<br>P06310 (IGKV2-30)<br>P02748 (C9)<br>P06331 (IGHV4-34)<br>P36980 (CFHR2)<br>P01859 (IGHG2)<br>P01780 (IGHV3-7)<br>P01764 (IGHV3-23)	15.34 (10 vs 0.65)<br>P04433 (IGKV3-11)<br>P01614 (IGKV2D-40)<br>P08246 (ELANE)<br>Q92496 (CFHR4)<br>P01817 (IGHV2-5)<br>P20851 (C4BPB)<br>P01611 (IGKV1D-12)<br>P15529 (CD46)<br>P01602 (IGKV1-5)<br>P15169 (CPN1)
Initial triggering of complement	9.57 (10 vs 1.04)<br>P04430 (IGKV1-16)<br>P80748 (IGLV3-21)<br>P01766 (IGHV3-13)<br>P01619 (IGKV3-20)<br>P06310 (IGKV2-30)<br>P06331 (IGHV4-34)<br>P01859 (IGHG2)<br>P48740 (MASP1)<br>P01780 (IGHV3-7)<br>P01764 (IGHV3-23)	11.91 (6 vs 0.50)<br>P04433 (IGKV3-11)<br>P01614 (IGKV2D-40)<br>P01817 (IGHV2-5)<br>P01611 (IGKV1D-12)<br>P01602 (IGKV1-5)<br>P01764 (IGHV3-23)
Complement cascade	9.49 (14 vs 1.47)	14.06 (10 vs 0.71)

	P04430 (IGKV1-16)<br>P22792 (CPN2)<br>P80748 (IGLV3-21)<br>P01766 (IGHV3-13)<br>P01031 (C5)<br>P01619 (IGKV3-20)<br>P06310 (IGKV2-30)<br>P02748 (C9)<br>P06331 (IGHV4-34)<br>P36980 (CFHR2)<br>P01859 (IGHG2)<br>P48740 (MASP1)<br>P01780 (IGHV3-7)<br>P01764 (IGHV3-23)	P04433 (IGKV3-11)<br>P01614 (IGKV2D-40)<br>P08246 (ELANE)<br>Q92496 (CFHR4)<br>P01817 (IGHV2-5)<br>P20851 (C4BPB)<br>P01611 (IGKV1D-12)<br>P15529 (CD46)<br>P01602 (IGKV1-5)<br>P15169 (CPN1)
Neutrophil degranulation	3.24 (19 vs 5.86)<br>P39656 (DDOST)<br>P37108 (SRP14)<br>P46940 (IQGAP1)<br>P05164 (MPO)<br>P30740 (SERPINB1)<br>Q9NZ32 (ACTR10)<br>P41218 (MNDA)<br>P06737 (PYGL)<br>P02766 (TTR)<br>P09429 (HMGB1)<br>Q86YZ3 (HRNR)<br>P04217 (A1BG)<br>P06702 (S100A9)<br>P24158 (PRTN3)<br>P05109 (S100A8)<br>P00738 (HP)<br>P14780 (MMP9)<br>P02763 (ORM1)<br>P02765 (AHSG)	5.31 (15 vs 2.83)<br>P07437 (TUBB)<br>P08311 (CTSG)<br>Q9P000 (COMMD9)<br>Q7Z3J2 (VPS35L)<br>P08246 (ELANE)<br>P08962 (CD63)<br>Q8N2G8 (GHDC)<br>P16671 (CD36)<br>P07355 (ANXA2)<br>P09429 (HMGB1)<br>P28799 (GRN)<br>P02775 (PPBP)<br>P04066 (FUCA1)<br>P68371 (TUBB4B)<br>P61586 (RHOA)
Platelet degranulation	6.41 (10 vs 1.56)<br>P00747 (PLG)<br>Q9BWS9 (CHID1)<br>Q96JJ7 (TMX3)<br>P49908 (SELENOP)<br>P04217 (A1BG)<br>P08697 (SERPINF2)<br>P08514 (ITGA2B)<br>P02763 (ORM1)<br>P01042 (KNG1)<br>P02765 (AHSG)	-
Platelet activation, signaling and aggregation	3.76 (12 vs 3.19)<br>P00747 (PLG)<br>Q9BWS9 (CHID1)<br>Q96JJ7 (TMX3)<br>P49908 (SELENOP)<br>P16885 (PLCG2)<br>P04217 (A1BG)<br>P12931 (SRC)<br>P08697 (SERPINF2)<br>P08514 (ITGA2B)<br>P02763 (ORM1)<br>P01042 (KNG1)<br>P02765 (AHSG)	-
Regulation of TLR by endogenous ligand	15.50 (4 vs 0.26)<br>P09429 (HMGB1)<br>P06702 (S100A9)<br>P04114 (APOB)<br>P05109 (S100A8)	-
Interferon signaling	3.72 (9 vs 2.42)<br>P19174 (PLCG1)	-

	P46934 (NEDD4)<br>Q9Y6K5 (OAS3)<br>Q8N1F7 (NUP93)<br>Q8IYM9 (TRIM22)<br>P29590 (PML)<br>P35658 (NUP214)<br>P01911 (HLA-DRB1)<br>P19525 (EIF2AK2)	
Innate immune system	3.23 (44 vs 13.60)<br>P08631 (HCK)<br>P19174 (PLCG1)<br>P39656 (DDOST)<br>P04430 (IGKV1-16)<br>P37108 (SRP14)<br>P35222 (CTNNB1)<br>P22792 (CPN2)<br>P46940 (IQGAP1)<br>Q96JJ3 (ELMO2)<br>P05164 (MPO)<br>P30740 (SERPINB1)<br>Q9NZ32 (ACTR10)<br>P80748 (IGLV3-21)<br>Q9NZQ3 (NCKIPSD)<br>P41218 (MNDA)<br>P01766 (IGHV3-13)<br>Q92556 (ELMO1)<br>P06737 (PYGL)<br>Q9Y6W5 (WASF2)<br>P01031 (C5)<br>P01619 (IGKV3-20)<br>P02766 (TTR)<br>P09429 (HMGB1)<br>P06310 (IGKV2-30)<br>Q86YZ3 (HRNR)<br>P02748 (C9)<br>P06331 (IGHV4-34)<br>P16885 (PLCG2)<br>P04217 (A1BG)<br>P06702 (S100A9)<br>P04114 (APOB)<br>P24158 (PRTN3)<br>P36980 (CFHR2)<br>P05109 (S100A8)<br>P00738 (HP)<br>P14780 (MMP9)<br>P01859 (IGHG2)<br>Q92530 (PSMF1)<br>P48740 (MASP1)<br>P02763 (ORM1)<br>P01780 (IGHV3-7)<br>P01764 (IGHV3-23)<br>P02765 (AHSG)<br>Q9NZC2 (TREM2)	3.96 (26 vs 6.56)<br>P07437 (TUBB)<br>P08311 (CTSG)<br>P04433 (IGKV3-11)<br>Q9P000 (COMMD9)<br>P01614 (IGKV2D-40)<br>Q7Z3J2 (VPS35L)<br>P08246 (ELANE)<br>P08962 (CD63)<br>Q8N2G8 (GHDC)<br>P16671 (CD36)<br>Q92496 (CFHR4)<br>P07355 (ANXA2)<br>P60953 (CDC42)<br>P01817 (IGHV2-5)<br>P09429 (HMGB1)<br>P20851 (C4BPB)<br>P01611 (IGKV1D-12)<br>P28799 (GRN)<br>P15529 (CD46)<br>P02775 (PPBP)<br>P50570 (DNM2)<br>P04066 (FUCA1)<br>P01602 (IGKV1-5)<br>P68371 (TUBB4B)<br>P15169 (CPN1)<br>P61586 (RHOA)
Cellular responses to stress	2.38 (21 vs 8.82)<br>Q9Y2L1 (DIS3)<br>P35611 (ADD1)<br>Q8N1F7 (NUP93)<br>P17096 (HMGA1)<br>Q9NZ32 (ACTR10)<br>P00403 (MT-CO2)<br>P16401 (H1-5)<br>P32119 (PRDX2)<br>Q86X55 (CARM1)<br>P04114 (APOB)<br>P27635 (RPL10)	-

	P42766 (RPL35)<br>P35658 (NUP214)<br>P26373 (RPL13)<br>P15880 (RPS2)<br>P46781 (RPS9)<br>Q99623 (PHB2)<br>P08240 (SRPRA)<br>Q92530 (PSMF1)<br>P40429 (RPL13A)<br>P07305 (H1-0)	
**Translation**		
Translation	3.33 (12 vs 3.60)<br>P39656 (DDOST)<br>P37108 (SRP14)<br>Q99613 (EIF3C)<br>Q9P2J5 (LARS1)<br>P27635 (RPL10)<br>P42766 (RPL35)<br>P26373 (RPL13)<br>P26639 (TARS1)<br>P15880 (RPS2)<br>P46781 (RPS9)<br>P08240 (SRPRA)<br>P40429 (RPL13A)	-
Eukaryotic translation initiation	4.79 (7 vs 1.46)<br>Q99613 (EIF3C)<br>P27635 (RPL10)<br>P42766 (RPL35)<br>P26373 (RPL13)<br>P15880 (RPS2)<br>P46781 (RPS9)<br>P40429 (RPL13A)	-
Eukaryotic translation elongation	5.19 (6 vs 1.16)<br>P27635 (RPL10)<br>P42766 (RPL35)<br>P26373 (RPL13)<br>P15880 (RPS2)<br>P46781 (RPS9)<br>P40429 (RPL13A)	-
Eukaryotic translation termination	5.25 (6 vs 1.14)<br>P27635 (RPL10)<br>P42766 (RPL35)<br>P26373 (RPL13)<br>P15880 (RPS2)<br>P46781 (RPS9)<br>P40429 (RPL13A)	-

Table S3. Distribution of proteins which are upregulated in either carotid atherosclerotic plaques or adjacent arterial segments (annotated according to the Gene Ontology Cellular Component database). DEPs: differentially expressed proteins.

GO term<br>(Cellular Component)	Carotid plaques<br>(fold change, observed versus expected numbers of DEPs)	Adjacent arterial segments<br>(fold change, observed versus expected numbers of DEPs)
**Cellular organelles**		
Platelet alpha granule	8.05 (9 vs 1.12)<br>P00747 (PLG)<br>Q96JJ7 (TMX3)<br>P04217 (A1BG)<br>P08697 (SERPINF2)<br>P08514 (ITGA2B)<br>P07093 (SERPINE2)<br>P02763 (ORM1)<br>P01042 (KNG1)<br>P02765 (AHSG)	-

Azurophil granule lumen	5.43 (6 vs 1.11)<br>P05164 (MPO)<br>Q9NZ32 (ACTR10)<br>P41218 (MNDA)<br>P02766 (TTR)<br>Q86YZ3 (HRNR)<br>P24158 (PRTN3)	13.13 (7 vs 0.53)<br>P07437 (TUBB)<br>P08311 (CTSG)<br>P08246 (ELANE)<br>P07355 (ANXA2)<br>P28799 (GRN)<br>P04066 (FUCA1)<br>P68371 (TUBB4B)
Ribosome	3.60 (10 vs 2.78)<br>P05090 (APOD)<br>P27635 (RPL10)<br>P42766 (RPL35)<br>Q9BRX2 (PELO)<br>P26373 (RPL13)<br>P15880 (RPS2)<br>P46781 (RPS9)<br>P19525 (EIF2AK2)<br>P40429 (RPL13A)<br>Q99613 (EIF3C)	-
Lysosome	2.75 (25 vs 9.11)<br>P08631 (HCK)<br>P39656 (DDOST)<br>Q96FZ7 (CHMP6)<br>Q9BWS9 (CHID1)<br>P05164 (MPO)<br>P30740 (SERPINB1)<br>Q9NZ32 (ACTR10)<br>Q9UN37 (VPS4A)<br>P41218 (MNDA)<br>P02766 (TTR)<br>Q9P2J5 (LARS1)<br>Q86YZ3 (HRNR)<br>P02774 (GC)<br>P04114 (APOB)<br>P24158 (PRTN3)<br>O00203 (AP3B1)<br>P12931 (SRC)<br>Q9Y2T2 (AP3M1)<br>P42892 (ECE1)<br>P01911 (HLA-DRB1)<br>Q9P2R3 (ANKFY1)<br>Q6IQ22 (RAB12)<br>Q6P9B6 (MEAK7)<br>P48426 (PIP4K2A)<br>Q5VIR6 (VPS53)	-
Endosome	2.28 (29 vs 12.69)<br>Q9UNF0 (PACSIN2)<br>Q96FZ7 (CHMP6)<br>Q9BWS9 (CHID1)<br>Q9NZN3 (EHD3)<br>Q5VIR6 (VPS53)<br>P30740 (SERPINB1)<br>Q96SU4 (OSBPL9)<br>Q9UN37 (VPS4A)<br>Q9NR09 (BIRC6)<br>Q9Y2I1 (NISCH)<br>Q9Y6W5 (WASF2)<br>Q6ZMI0 (PPP1R21)<br>P09429 (HMGB1)<br>P29590 (PML)<br>Q92783 (STAM)<br>Q8IWB7 (WDFY1)<br>P04114 (APOB)<br>Q9Y296 (TRAPPC4)<br>P50281 (MMP14)	-

	O00203 (AP3B1)<br>Q99961 (SH3GL1)<br>P12931 (SRC)<br>Q92572 (AP3S1)<br>Q9Y2T2 (AP3M1)<br>P42892 (ECE1)<br>P01911 (HLA-DRB1)<br>Q9P2R3 (ANKFY1)<br>Q6IQ22 (RAB12)<br>Q8IYI6 (EXOC8)	
**Extracellular vesicles**		
Secretory granule lumen	5.09 (20 vs 3.93)<br>P37108 (SRP14)<br>P00747 (PLG)<br>P05164 (MPO)<br>P30740 (SERPINB1)<br>Q9NZ32 (ACTR10)<br>P41218 (MNDA)<br>P06737 (PYGL)<br>P49908 (SELENOP)<br>P02766 (TTR)<br>P09429 (HMGB1)<br>Q86YZ3 (HRNR)<br>P04217 (A1BG)<br>P06702 (S100A9)<br>P24158 (PRTN3)<br>P05109 (S100A8)<br>P08697 (SERPINF2)<br>P00738 (HP)<br>P02763 (ORM1)<br>P01042 (KNG1)<br>P02765 (AHSG)	6.33 (12 vs 1.90)<br>P07437 (TUBB)<br>P08311 (CTSG)<br>Q9P000 (COMMD9)<br>P08246 (ELANE)<br>Q8N2G8 (GHDC)<br>P07355 (ANXA2)<br>P09429 (HMGB1)<br>P28799 (GRN)<br>P02775 (PPBP)<br>P04066 (FUCA1)<br>P68371 (TUBB4B)<br>P35625 (TIMP3)
Extracellular vesicle	2.91 (76 vs 26.09)<br>Q9UNF0 (PACSIN2)<br>P46934 (NEDD4)<br>P00747 (PLG)<br>P43652 (AFM)<br>P35222 (CTNNB1)<br>P22792 (CPN2)<br>Q96FZ7 (CHMP6)<br>P46940 (IQGAP1)<br>Q9BWS9 (CHID1)<br>P04278 (SHBG)<br>P05164 (MPO)<br>Q6ZMP0 (THSD4)<br>Q96FV2 (SCRN2)<br>P30740 (SERPINB1)<br>P05090 (APOD)<br>P01019 (AGT)<br>P80748 (IGLV3-21)<br>Q9Y3C8 (UFC1)<br>E9PAV3 (NACA)<br>P00739 (HPR)<br>Q9UN37 (VPS4A)<br>Q9BPX5 (ARPC5L)<br>P08729 (KRT7)<br>P41218 (MNDA)<br>P30536 (TSPO)<br>P05091 (ALDH2)<br>P46926 (GNPDA1)<br>P06737 (PYGL)<br>Q9Y6W5 (WASF2)<br>P01031 (C5)<br>P01619 (IGKV3-20)<br>P49908 (SELENOP)	3.10 (39 vs 12.58)<br>P50440 (GATM)<br>P46379 (BAG6)<br>P48723 (HSPA13)<br>P07437 (TUBB)<br>P08311 (CTSG)<br>P04433 (IGKV3-11)<br>P01614 (IGKV2D-40)<br>Q9UBX5 (FBLN5)<br>P08246 (ELANE)<br>Q9UGM5 (FETUB)<br>P08962 (CD63)<br>P04180 (LCAT)<br>Q96FN4 (CPNE2)<br>Q96CW1 (AP2M1)<br>P07355 (ANXA2)<br>P60953 (CDC42)<br>Q6V0I7 (FAT4)<br>Q9Y6W3 (CAPN7)<br>P01611 (IGKV1D-12)<br>P29373 (CRABP2)<br>P28799 (GRN)<br>Q96NY7 (CLIC6)<br>Q99805 (TM9SF2)<br>P15529 (CD46)<br>P50570 (DNM2)<br>P04066 (FUCA1)<br>P01602 (IGKV1-5)<br>P19440 (GGT1)<br>P15311 (EZR)<br>P31689 (DNAJA1)<br>Q9UGV2 (NDRG3)<br>P68371 (TUBB4B)

	P02766 (TTR)<br>P06310 (IGKV2-30)<br>Q86YZ3 (HRNR)<br>P02748 (C9)<br>P32119 (PRDX2)<br>Q9UKV8 (AGO2)<br>Q9UK55 (SERPINA10)<br>Q9UKK3 (PARP4)<br>P16885 (PLCG2)<br>P02774 (GC)<br>P04217 (A1BG)<br>Q92688 (ANT32B)<br>P21589 (NT5E)<br>P06702 (S100A9)<br>Q8TBX8 (PIP4K2C)<br>P04114 (APOB)<br>P16870 (CPE)<br>P24158 (PRTN3)<br>O00203 (AP3B1)<br>P12931 (SRC)<br>P05109 (S100A8)<br>P42892 (ECE1)<br>P27169 (PON1)<br>P01911 (HLA-DRB1)<br>Q9P2R3 (ANKFY1)<br>P26639 (TARS1)<br>P08697 (SERPINF2)<br>P15880 (RPS2)<br>P46781 (RPS9)<br>P00738 (HP)<br>P08514 (ITGA2B)<br>P08240 (SRPRA)<br>P07093 (SERPINE2)<br>Q9P265 (DIP2B)<br>P14780 (MMP9)<br>P01859 (IGHG2)<br>P02763 (ORM1)<br>P01780 (IGHV3-7)<br>P01042 (KNG1)<br>P04908 (H2AC8)<br>P15104 (GLUL)<br>P01764 (IGHV3-23)<br>P02765 (AHSG)<br>P00966 (ASS1)	Q99985 (SEMA3C)<br>P47895 (ALDH1A3)<br>Q9Y263 (PLAA)<br>P61586 (RHOA)<br>P32189 (GK)<br>P01764 (IGHV3-23)<br>P30711 (GSTT1)
Secretory granule	2.79 (30 vs 10.75)<br>P39656 (DDOST)<br>P37108 (SRP14)<br>P00747 (PLG)<br>P46940 (IQGAP1)<br>P05164 (MPO)<br>P30740 (SERPINB1)<br>Q9NZ32 (ACTR10)<br>Q96JJ7 (TMX3)<br>P41218 (MNDA)<br>A1A4V9 (CFAP119)<br>P06737 (PYGL)<br>P49908 (SELENOP)<br>P02766 (TTR)<br>P09429 (HMGB1)<br>Q86YZ3 (HRNR)<br>P04217 (A1BG)<br>P06702 (S100A9)<br>P16870 (CPE)<br>P24158 (PRTN3)<br>P05109 (S100A8)	3.47 (18 vs 5.18)<br>P07437 (TUBB)<br>P08311 (CTSG)<br>Q9P000 (COMMD9)<br>Q7Z3J2 (VPS35L)<br>P08246 (ELANE)<br>P08962 (CD63)<br>Q8N2G8 (GHDC)<br>P16671 (CD36)<br>P07355 (ANXA2)<br>P60953 (CDC42)<br>P09429 (HMGB1)<br>P28799 (GRN)<br>P15529 (CD46)<br>P02775 (PPBP)<br>P04066 (FUCA1)<br>P68371 (TUBB4B)<br>P61586 (RHOA)<br>P35625 (TIMP3)

	P42892 (ECE1)<br>P08697 (SERPINF2)<br>P00738 (HP)<br>P08514 (ITGA2B)<br>P07093 (SERPINE2)<br>P14780 (MMP9)<br>P02763 (ORM1)<br>P01042 (KNG1)<br>P02765 (AHSG)<br>Q9Y296 (TRAPPC4)	
Secretory vesicle	2.50 (32 vs 12.82)<br>P39656 (DDOST)<br>P37108 (SRP14)<br>P00747 (PLG)<br>P46940 (IQGAP1)<br>P05164 (MPO)<br>P30740 (SERPINB1)<br>Q9NZ32 (ACTR10)<br>Q96JJ7 (TMX3)<br>P41218 (MNDA)<br>A1A4V9 (CFAP119)<br>P06737 (PYGL)<br>P49908 (SELENOP)<br>P02766 (TTR)<br>P09429 (HMGB1)<br>Q86YZ3 (HRNR)<br>P04217 (A1BG)<br>P06702 (S100A9)<br>P16870 (CPE)<br>Q9Y296 (TRAPPC4)<br>P24158 (PRTN3)<br>P05109 (S100A8)<br>P42892 (ECE1)<br>P08697 (SERPINF2)<br>P00738 (HP)<br>P08514 (ITGA2B)<br>P07093 (SERPINE2)<br>Q6IQ22 (RAB12)<br>P14780 (MMP9)<br>P02763 (ORM1)<br>P01042 (KNG1)<br>Q8TDW5 (SYTL5)<br>P02765 (AHSG)	2.91 (18 vs 6.18)<br>P07437 (TUBB)<br>P08311 (CTSG)<br>Q9P000 (COMMD9)<br>Q7Z3J2 (VPS35L)<br>P08246 (ELANE)<br>P08962 (CD63)<br>Q8N2G8 (GHDC)<br>P16671 (CD36)<br>P07355 (ANXA2)<br>P60953 (CDC42)<br>P09429 (HMGB1)<br>P28799 (GRN)<br>P15529 (CD46)<br>P02775 (PPBP)<br>P04066 (FUCA1)<br>P68371 (TUBB4B)<br>P61586 (RHOA)<br>P35625 (TIMP3)
**Extracellular compartment**		
Immunoglobulin complex, circulating	6.93 (8 vs 1.16)<br>P04430 (IGKV1-16)<br>P80748 (IGLV3-21)<br>P01766 (IGHV3-13)<br>P01619 (IGKV3-20)<br>P06310 (IGKV2-30)<br>P06331 (IGHV4-34)<br>P01859 (IGHG2)<br>P01764 (IGHV3-23)	17.95 (10 vs 0.56)<br>P04433 (IGKV3-11)<br>P01614 (IGKV2D-40)<br>A0A0C4DH36 (IGHV3-38)<br>A0A0B4J1U7 (IGHV6-1)<br>P01817 (IGHV2-5)<br>A0A0J9YX35 (IGHV3-64D)<br>A0A087WSZ0 (IGKV1D-8)<br>P01611 (IGKV1D-12)<br>P01602 (IGKV1-5)<br>P01764 (IGHV3-23)
Immunoglobulin complex	5.19 (12 vs 2.31)<br>P04430 (IGKV1-16)<br>A0A0C4DH38 (IGHV5-51)<br>P80748 (IGLV3-21)<br>P01766 (IGHV3-13)<br>P01619 (IGKV3-20)<br>A0A075B6J9 (IGLV2-18)<br>P06310 (IGKV2-30)<br>P06331 (IGHV4-34)	15.26 (17 vs 1.11)<br>P04433 (IGKV3-11)<br>P01614 (IGKV2D-40)<br>A0A0C4DH36 (IGHV3-38)<br>A0A0B4J1U7 (IGHV6-1)<br>P01817 (IGHV2-5)<br>A0A0J9YX35 (IGHV3-64D)<br>A0A087WSZ0 (IGKV1D-8)<br>P01611 (IGKV1D-12)

	P01859 (IGHG2)<br>A0A0A0MS15 (IHGV3-49)<br>P01780 (IGHV3-7)<br>P01764 (IGHV3-23)	A0A0C4DH34 (IGHV4-28)<br>P01602 (IGKV1-5)<br>A0A0B4J1X5 (IGHV3-74)<br>A0A0B4J1U3 (IGLV1-36)<br>A0A0C4DH35 (IGHV3-35)<br>A0A0A0MT36 (IGKV6D-21)<br>A0A0B4J1V2 (IGHV2-26)<br>A0A0A0MS15 (IGHV3-49)<br>P01764 (IGHV3-23)
Extracellular matrix	2.99 (21 vs 7.03)<br>P00747 (PLG)<br>P22792 (CPN2)<br>Q6UY14 (ADAMTSL4)<br>Q6ZMP0 (THSD4)<br>P30740 (SERPINB1)<br>P01019 (AGT)<br>P19022 (CDH2)<br>Q86YZ3 (HRNR)<br>P08709 (F7)<br>P04217 (A1BG)<br>P06702 (S100A9)<br>P50281 (MMP14)<br>P24158 (PRTN3)<br>P05109 (S100A8)<br>P08697 (SERPINF2)<br>P07093 (SERPINE2)<br>P14780 (MMP9)<br>P02763 (ORM1)<br>P01042 (KNG1)<br>P02765 (AHSG)<br>P35858 (IGFALS)	-

Table S4. Molecular functions of proteins which are upregulated in either carotid atherosclerotic plaques or adjacent arterial segments (annotated according to the Gene Ontology Molecular Function database). DEPs: differentially expressed proteins.

GO term (Molecular Function)	Carotid plaques (fold change, observed versus expected numbers of DEPs)	Adjacent arterial segments (fold change, observed versus expected numbers of DEPs)
**Cellular organelles**		
NF-κB binding	12.72 (5 vs 0.39)<br>Q92769 (HDAC2)<br>Q7Z4G1 (COMMD6)<br>Q9UNN5 (FAF1)<br>Q96JB5 (CDK5RAP3)<br>Q86UE4 (MTDH)	-
Translation regulator activity	4.92 (9 vs 1.83)<br>Q99613 (EIF3C)<br>Q6PKG0 (LARP1)<br>Q9UKV8 (AGO2)<br>Q8NE71 (ABCF1)<br>P27635 (RPL10)<br>P42766 (RPL35)<br>P26373 (RPL13)<br>P15880 (RPS2)<br>P46781 (RPS9)	-
Transcription coactivator activity	3.76 (13 vs 3.45)<br>Q96PK6 (RBM14)<br>P35222 (CTNNB1)<br>P17096 (HMGA1)<br>E9PAV3 (NACA)	-

	Q9Y2Y0 (ARL2BP)<br>Q8IYM9 (TRIM22)<br>P09429 (HMGB1)<br>Q86X55 (CARM1)<br>Q969T9 (WBP2)<br>Q9BUP3 (HTATIP2)<br>Q86UE4 (MTDH)<br>Q99417 (MYCBP)<br>P02766 (TTR)	
Lipid binding	2.45 (24 vs 9.86)<br>P08631 (HCK)<br>Q9UNF0 (PACSIN2)<br>P46940 (IQGAP1)<br>P04278 (SHBG)<br>P05090 (APOD)<br>Q9BR39 (JPH2)<br>Q96SU4 (OSBPL9)<br>Q9Y2I1 (NISCH)<br>P30536 (TSPO)<br>P09429 (HMGB1)<br>P02774 (GC)<br>Q92783 (STAM)<br>P06702 (S100A9)<br>Q8IWB7 (WDFY1)<br>P04114 (APOB)<br>Q99961 (SH3GL1)<br>P05109 (S100A8)<br>P27169 (PON1)<br>Q9P2R3 (ANKFY1)<br>Q63HR2 (TNS2)<br>Q99623 (PHB2)<br>Q9UPT5 (EXOC7)<br>O00291 (HIP1)<br>Q8TDW5 (SYTL5)	-
